# Risk of acute respiratory infection and acute cardiovascular events following acute respiratory infection among adults with increased cardiovascular risk in England between 2008 and 2018: a retrospective, population-based cohort study

**DOI:** 10.1016/S2589-7500(21)00203-X

**Published:** 2021-11-22

**Authors:** Jennifer A Davidson, Amitava Banerjee, Liam Smeeth, Helen I McDonald, Daniel Grint, Emily Herrett, Harriet Forbes, Richard Pebody, Charlotte Warren-Gash

**Affiliations:** a Department of Non-Communicable Disease Epidemiology, London School of Hygiene & Tropical Medicine, London, UK; b Department of Infectious Disease Epidemiology, London School of Hygiene & Tropical Medicine, London, UK; c National Institute for Health Research Health Protection Research Unit in Immunisation, London School of Hygiene & Tropical Medicine, London, UK; d Institute of Health Informatics, University College London, London, UK; e Population Health Sciences, Bristol Medical School, University of Bristol, Bristol, UK; f National Infection Service, Public Health England, London, UK

## Abstract

**Background:**

Although acute respiratory infections can lead to cardiovascular complications, the effect of underlying cardiovascular risk on the incidence of acute respiratory infections and cardiovascular complications following acute respiratory infection in individuals without established cardiovascular disease is unknown. We aimed to investigate whether cardiovascular risk is associated with increased risk of acute respiratory infection and acute cardiovascular events after acute respiratory infection using 10 years of linked electronic health record (EHR) data in England.

**Methods:**

In this retrospective, population-based cohort study we used EHRs from primary care providers registered on the Clinical Practice Research Datalink (CPRD) GOLD and Aurum databases in England. Eligible individuals were aged 40–64 years, did not have established cardiovascular disease or a chronic health condition that would make them eligible for influenza vaccination, were registered at a general practice contributing to the CPRD, and had linked Hospital Episode Statistics Admitted Patient Care data in England from Sept 1, 2008, to Aug 31, 2018. We classified cardiovascular risk on the basis of diagnosed hypertension and overall predicted cardiovascular risk, estimated by use of the QRISK2 risk-prediction tool (comparing a score of ≥10% [increased risk] with a score of <10% [low risk]). Using multivariable Poisson regression models, we calculated incidence rate ratios (IRRs) for systemic acute respiratory infection. Among individuals who had an acute respiratory infection, we used multivariable Cox regression to calculate hazard ratios (HRs) for the risk of acute cardiovascular events within 1 year of infection.

**Findings:**

We identified 6 075 321 individuals aged 40–64 years with data in the CPRD and linked data in the Hospital Episode Statistics Admitted Patient Care database between Sept 1, 2008, and Aug 31, 2018. Of these individuals, 4 212 930 (including 526 480 [12·5%] with hypertension and 607 087 [14·4%] with a QRISK2 score of ≥10%) were included in the assessment of the incidence of acute respiratory infection. After adjusting for confounders (age, sex, ethnicity, socioeconomic status, body-mass index, alcohol consumption, smoking status, and consultation frequency in the hypertension analysis; and alcohol consumption and consultation frequency in the QRISK2 analysis), the incidence of acute respiratory infection was higher in individuals with hypertension than those without (IRR 1·04 [95% CI 1·03–1·05]) and higher in those with a QRISK2 score of 10% or higher than in those with a QRISK2 score of less than 10% (1·39 [1·37–1·40]). Of the 442 408 individuals who had an acute respiratory infection, 4196 (0·9%) had an acute cardiovascular event within 1 year of infection. After adjustment (for age, sex, ethnicity, socioeconomic status, body-mass index, alcohol consumption, and smoking status in the hypertension analysis; and for alcohol consumption in the QRISK2 analysis), hypertension (HR 1·98 [95% CI 1·83–2·15]) and a QRISK2 score of 10% or higher (3·65 [3·42–3·89]) were associated with a substantially increased risk of acute cardiovascular events after acute respiratory infection.

**Interpretation:**

People with increased cardiovascular risk but without diagnosed cardiovascular disease, measured by diagnosed hypertension or overall predicted cardiovascular risk, could benefit from influenza and pneumococcal vaccine prioritisation to reduce their risk of both acute respiratory infection and cardiovascular complications following an acute respiratory infection.

**Funding:**

British Heart Foundation and the Wellcome Trust.

## Introduction

The COVID-19 pandemic has expedited research on cardiovascular complications following systemic acute respiratory infection. Before the pandemic, observational studies showed that acute respiratory infections increased the risk of myocardial infarction and stroke. In self-controlled case-series using large electronic health record (EHR) datasets, risk of myocardial infarction and stroke was elevated by 2–6 times in the days following clinically diagnosed acute respiratory infection, with the risk remaining elevated for up to 1 month.^1^, ^2^ A range of organisms, including *Streptococcus pneumoniae* and the influenza virus, are known to trigger cardiovascular events.^3^, ^4^

**Research in context**

**Evidence before this study**

Before the COVID-19 pandemic, self-controlled case-series estimated a 2–6 times transient increase in the risk of myocardial infarction and stroke following a range of clinically diagnosed or laboratory-confirmed acute respiratory infections. These studies did not explore whether these effects were modified by underlying cardiovascular risk. We searched PubMed using the search terms “*hypertensi*” OR “cardiovascular risk” AND “influenza” OR “pneumonia” OR “respiratory infection” AND “cardiovascular event” OR “myocardial infarction” OR “acute coronary syndrome” OR “stroke” OR “heart failure” for primary research studies and reviews investigating the effect of underlying cardiovascular risk on cardiovascular complications after acute respiratory infection published in any language, from database inception to Jan 25, 2021. Of 421 studies identified, three presented estimates for the effect of cardiovascular risk on cardiovascular complications after acute respiratory infection. One prospective cohort study of individuals with community-acquired pneumonia from five North American medical centres in 1991–94 showed that arterial hypertension was associated with an increased odds of cardiac complications (new or worsening heart failure, new or worsening arrhythmias, or myocardial infarction) within 30 days of a diagnosis of community-acquired pneumonia. The second study used clinical records from Beijing, China, to explore risk factors for cardiovascular complications after hospitalisation for community-acquired pneumonia in 2013–15, and found that patients hospitalised with community-acquired pneumonia who had cardiovascular complications had a significantly higher prevalence of hypertension than those without cardiovascular complications. The final study used UK primary care electronic health records (EHRs) to identify individuals who had a myocardial infarction or stroke in 1995–2004 and matched (on year of birth, sex, primary care practice, and calendar time) controls. The authors found that individuals who had an acute respiratory infection in the previous month had an increased odds of having a first myocardial infarction or stroke, regardless of background cardiovascular risk. The effect of underlying cardiovascular risk on cardiovascular complications after acute respiratory infection therefore remains unclear. We also did a search of PubMed on Sept 28, 2021, using the search terms “*hypertensi*” OR “cardiovascular risk” AND “influenza” OR “pneumonia” OR “respiratory infection”. We searched for research articles or reviews published in any language between database inception and Jan 1, 2021. Of the 2175 studies identified, one presented estimates for the effect that cardiovascular risk has on the incidence of acute respiratory infections itself. The study used UK Biobank data to show that hypertension was independently associated with an increased risk of acute lower respiratory infections, particularly pneumonia.

**Added value of this study**

Using primary and secondary care EHRs from more than 4·2 million individuals in England, we found that the incidence of acute respiratory infection, particularly pneumonia, was higher in those with increased cardiovascular risk, defined as diagnosed hypertension or a QRISK2 10-year risk score of 10% or more. In addition, individuals with increased cardiovascular risk also had an elevated risk of acute cardiovascular events after an acute respiratory infection. This risk was more pronounced when QRISK2 score, which incorporates multiple factors associated with cardiovascular disease onset, was used rather than a diagnosis of hypertension alone. Therefore, QRISK2 provides a practical method to identify individuals at risk of cardiovascular complications after acute respiratory infection and to thereby prevent early-onset cardiovascular disease.

**Implications of all the available evidence**

Our analyses and the available evidence to date indicate that individuals with increased cardiovascular risk have a higher risk of acute respiratory infection, and that these infections are more likely to trigger acute cardiovascular events than in those without increased cardiovascular risk. These findings highlight the importance of managing and reducing cardiovascular risk to reduce acute respiratory infections and their consequences. Individuals with increased cardiovascular risk have not been considered at high risk of SARS-CoV-2 infection or COVID-19 complications in policy decisions; however, hypertension has been associated with an increased risk of severe outcomes in most analyses. Individuals with increased cardiovascular risk are not typically targeted for seasonal influenza or pneumococcal vaccines, and have not been prioritised in the roll-out of COVID-19 vaccines. The prevention or treatment of acute respiratory infection in individuals with increased cardiovascular risk, as well as in those with established cardiovascular disease, could reduce the risk of cardiovascular events.

Observational studies and the few randomised controlled trials (RCTs) published to date show that pneumococcal and influenza vaccines reduce the risk of cardiovascular complications when compared with placebo or no vaccine.^5^, ^6^, ^7^ A meta-analysis of secondary prevention RCTs found that influenza vaccination reduced cardiovascular mortality by 55%.^7^ Pneumococcal vaccination reduced the odds of acute coronary syndrome by 17% in a meta-analysis of observational studies conducted among individuals aged 65 years or older.^5^

In Europe, people with underlying cardiovascular risk but without established cardiovascular disease are not typically recommended to receive seasonal influenza or pneumococcal vaccines.^8^, ^9^, ^10^ Public Health England recommends a one-time polysaccharide pneumococcal vaccine and seasonal influenza vaccines for adults aged 65 years or older and for those aged younger than 65 years with specific underlying health conditions, including chronic heart disease.^9^, ^10^

An association between high blood pressure and cardiovascular complications has been observed in individuals as young as 40 years of age.^11^ Blood pressure is an essential predictor of cardiovascular risk, but it is only one component. Cardiovascular risk scores such as the QRISK2^12^ are increasingly used to predict an individual's likelihood, most commonly the 10-year risk, of future cardiovascular disease on the basis of multiple factors. The National Institute for Health and Care Excellence recommends the use of the QRISK2 score for assessment and management of cardiovascular disease.^13^

Little is known about the effect of cardiovascular risk on the risk of acute respiratory infection; however, a recent UK Biobank study showed that high blood pressure increased the risk of acute respiratory infection, particularly pneumonia and influenza.^14^ Similarly, the role of cardiovascular risk in the association between acute respiratory infection and cardiovascular complications has only been sporadically studied. We identified two previous studies that reported an association between hypertension and cardiovascular complications after pneumonia.^15^, ^16^ Another previous study investigated the association between overall cardiovascular risk and myocardial infarction and stroke after acute respiratory infection.^17^ This previous study reported an increased risk of myocardial infarction and stroke after acute respiratory infection, regardless of cardiovascular risk level. Cardiovascular risk was derived from associations identified in univariable analysis (angina, hypertension, hyperlipidaemia, diabetes, chronic heart disease among first degree relatives, peripheral vascular disease, smoking status, and previous stroke for myocardial infarction, and hypertension, diabetes, peripheral vascular disease, smoking status, previous myocardial infarction, and urinary tract infection for stroke) rather than a pre-existing risk score.^17^

In this study, we aimed to investigate how cardiovascular risk, defined by diagnosed hypertension or QRISK2 score, is associated with the risk of acute respiratory infection and acute cardiovascular events after acute respiratory infection using 10 years of linked EHR data in England.

## Methods

### Study design and data sources

We did a retrospective cohort study using routinely collected primary care data from the Clinical Practice Research Datalink (CPRD). The CPRD collects anonymised coded data from general practices in England through the GOLD database using the Vision clinical management system and Aurum database using the EMIS clinical management system.^18^, ^19^ The databases include more than 35 million individuals, representative of the UK population in terms of age, sex, and ethnicity. The coded data collected include diagnoses, prescriptions, immunisations, and basic demographic characteristics.

Consenting general practices have patient records linked to other data sources. We used linked secondary care data from the Hospital Episode Statistics Admitted Patient Care (HES APC) database, data on deaths from the Office for National Statistics (ONS), and individual-level Townsend deprivation index scores. HES APC data contain information on diagnoses made and procedures done for all National Health Service (NHS) admissions in England, coded with the International Classification of Diseases version 10 (ICD-10).^20^ The ONS mortality data contain information on the date and cause of death, also coded with the ICD-10.

The CPRD Independent Scientific Advisory Committee approved this study (19_209). The CPRD provided data on relevant HES APC, ONS, and Townsend index variables for the study population. The London School of Hygiene & Tropical Medicine provided ethical approval (17894).

The study protocol and data analysis plan are available in the appendix (pp 21–31).

### Participants

Eligible individuals were aged 40–64 years, not already recommended for seasonal influenza vaccination according to current UK guidelines,^9^ and registered at a general practice contributing to the CPRD (via GOLD or Aurum) with linked HES APC data in England from Sept 1, 2008, to Aug 31, 2018. This time period covered the duration of QRISK2 use. We started follow-up in September as this is when primary care practices identify individuals eligible for the seasonal influenza vaccine. We selected individuals aged 40–64 years to include those who had an increased likelihood of having a high cardiovascular risk or acute cardiovascular events, or both, but who were younger than the age cutoff for universal influenza vaccination. We defined the start of follow-up as Sept 1, 2008, the individual's 40th birthday, or the research standard CPRD date (12 months after current registration in the Aurum dataset, and the latest of 12 months after the current registration or the practice research standard in the GOLD dataset), whichever came first.

We excluded individuals who had established cardiovascular disease, received a previous pneumococcal vaccine, received an influenza vaccine within the previous 12 months, or a chronic health condition making them eligible for influenza vaccination, recorded in the CPRD at baseline. We defined conditions recommended for influenza vaccination as chronic liver disease, chronic respiratory disease, stage 3–5 chronic kidney disease, diabetes, asplenia or other splenic dysfunction, chronic neurological conditions, severe obesity (ie, a body-mass index [BMI] of ≥40 kg/m^2^), or an immunosuppressive condition.^9^ Full exclusion criteria are provided in the appendix (p 2).

Individual patient consent was not required to collect the original underlying data because data were generated during routine clinical encounters. Consent was given at the general practice level for anonymised primary care data to be used for research through contributing to the CPRD. Patients can advise their general practice if they wish to opt out of data collection. For our research we have obtained the data from the CPRD following their approval process.

### Outcomes and variables

We used the whole study population to investigate the incidence of acute respiratory infection. We ended follow-up at diagnosis of cardiovascular disease or a chronic condition with which an individual would be recommended for influenza vaccination, receipt of pneumococcal or influenza vaccination, death, transfer out of general practice, the date of last data collection from general practice, the individual's 65th birthday, or Aug 31, 2018, whichever came first (appendix p 3).

We defined acute respiratory infection as a clinical or confirmed diagnosis of pneumonia, acute bronchitis, influenza, influenza-like illness, or other acute infections suggestive of lower respiratory tract involvement in the CPRD or HES APC record. Selected diagnostic codes were based on the code lists used in a previous study.^2^ We did not include symptoms in our definition. In further analyses, we analysed the incidence of influenza or influenza-like illness and pneumonia separately. For each individual, we grouped acute respiratory infection records within 28 days into a single episode (see appendix [p 3] for full details).

Within the study population, individuals with an acute respiratory infection were followed up from diagnosis to investigate the incidence and risk of acute cardiovascular events after this infection. Follow-up for this study population ended at the occurrence of an acute cardiovascular event, death, transfer out of general practice, the date of last data collection from general practice, 1 year after diagnosis of the acute respiratory infection, or Aug 31, 2018, whichever came first (appendix p 3).

For our main analysis, we used a broad definition for acute cardiovascular events of acute coronary syndrome (myocardial infarction and unstable angina), left ventricular heart failure, stroke or transient ischaemic attack, acute limb ischaemia, or cardiovascular death. We included diagnoses recorded in the CPRD or HES APC, with the codes used informed by previous studies,^21^ and cardiovascular deaths (ICD-10 codes I00–I99) recorded by the ONS. In further analyses, we also assessed each cardiovascular condition separately.

We considered two measures of cardiovascular risk: diagnosed hypertension and QRISK2 score. QRISK2 is a prediction algorithm that estimates an individual's 10-year risk of cardiovascular disease.^12^ The risk factors included and the process we used to calculate QRISK2 scores are outlined in the appendix (p 4). Briefly, a score is calculated using a range of risk factors, such as age, sex, ethnicity, socioeconomic status, comorbid health conditions, BMI, blood pressure reading, and smoking status. QRISK2 is not widely used outside the UK; therefore, we also included hypertension as a pragmatic definition of cardiovascular risk. To ensure that we included only individuals with persistent and diagnosed hypertension, we used coded CPRD diagnoses with no time limit.

Individuals were classified as having increased cardiovascular risk (ie, diagnosed hypertension or a QRISK2 score of ≥10%) or not (no hypertension or a QRISK2 score of <10%) at baseline, and their risk was updated if hypertension was recorded or new measures relevant to the QRISK2 algorithm resulted in a change in QRISK2 score from less than 10% to 10% or higher during follow-up.

We considered demographics, lifestyle factors, and primary care consultation frequency in analyses of hypertension and acute respiratory infection. The demographic features were age (5-year bands of 40–44, 45–49, 50–54, 55–59, and 60–64 years), sex (male and female), race or ethnicity (White, Black, south Asian, and mixed or other), and socioeconomic status (individual-level Townsend score data grouped into quintiles, ranging from least deprived [quintile 1] to most deprived [quintile 5]). The lifestyle factors were baseline alcohol consumption (heavy drinking [defined as either a recorded intake of >42 units per week or a diagnostic code suggestive of alcohol addiction or excessive alcohol consumption] or no known heavy drinking), smoking status (current smoker, never smoker, or former smoker), and BMI (underweight [BMI <18·5 kg/m^2^], normal [BMI 18·5–24·9 kg/m^2^], overweight [BMI 25·0–29·9 kg/m^2^], or obese [BMI 30·0–39·9 kg/m^2^]). Consultation frequency was derived from the number of in-person or telephone consultations in the year before baseline. For analyses involving QRISK2 scores, we included only alcohol consumption and consultation frequency, as all other factors are included in the QRISK2 algorithm. We identified these included covariates using CPRD data, with data on race or ethnicity additionally collected from the HES APC database.

Our analyses of acute cardiovascular events after acute respiratory infection included the aforementioned demographic and lifestyle factors. We stratified results by statin, antihypertensive, or antiplatelet prescriptions recorded in the CPRD in the year before infection. Our rationale for covariate and effect-modifier selection is explained in the appendix (p 4).

### Statistical analysis

We pooled individual-level data from the CPRD GOLD and Aurum databases. We calculated age-stratified acute respiratory infection incidence rates by cardiovascular risk. We used multivariable Poisson regression to calculate incidence rate ratios (IRRs). To account for multiple episodes of acute respiratory infection per individual, the random-effects models included time-updated exposure by age and cardiovascular risk. We initially adjusted for age and sex before adjusting for additional covariates (age, sex, ethnicity, socioeconomic status, BMI, alcohol intake, smoking status, and consultation frequency in the hypertension analysis; and alcohol consumption and consultation frequency in the QRISK2 analysis). A complete case-analysis approach was used in multivariable analyses. We did not do multiple imputation because data in CPRD are unlikely to be missing at random. Among individuals with an acute cardiovascular event within 1 year of acute respiratory infection, we summarised (using medians [IQRs]) the time between the infection and event. To assess the effect of cardiovascular risk on acute cardiovascular events after acute respiratory infection, we used multivariable Cox proportional hazards regression finely adjusted for time under follow-up. We used robust standard errors and initially adjusted for age and sex before adjusting for additional covariates (age, sex, ethnicity, socioeconomic status, BMI, alcohol consumption, and smoking status in the hypertension analysis; and alcohol intake in the QRISK2 analysis). In all analyses, for individuals who entered and exited follow-up on the same day, we added 1 day to ensure all individuals contributed at least 1 day of follow-up. Additionally, we separately added an interaction term to our Cox random-effects models for statin, antihypertensive, or antiplatelet prescriptions, and compared the results to models without interaction.

We did five prespecified sensitivity analyses. First, we validated results obtained from combined individual-level GOLD and Aurum data by analysing each database separately before combining them with a random-effects meta-analysis. Between-database heterogeneity was assessed by use of the *I*^2^ statistic. Second, we excluded only individuals eligible for both pneumococcal and influenza vaccines (see appendix p 2).^9^, ^10^ Third, we repeated the analyses restricted to individuals with a QRISK2 score recorded by their general practitioner and collected in the CPRD from Jan 1, 2015, to Dec 31, 2017 (see appendix p4 for further detail of why this restricted time period was used), to compare results with those of our calculated QRISK2 scores and ensure consistent results were obtained. Fourth, we included only major adverse cardiovascular events of myocardial infarction, heart failure, stroke, and cardiovascular death in the cardiovascular event outcome. Finally, we repeated the analysis of acute cardiovascular events after acute respiratory infection excluding individuals who received pneumococcal or influenza vaccines during follow-up.

All statistical analyses were done using Stata, version 16.

### Role of the funding source

The funders of the study had no role in study design, data collection, data analysis, data interpretation, or writing of the report.

## Results

**Figure 1:**
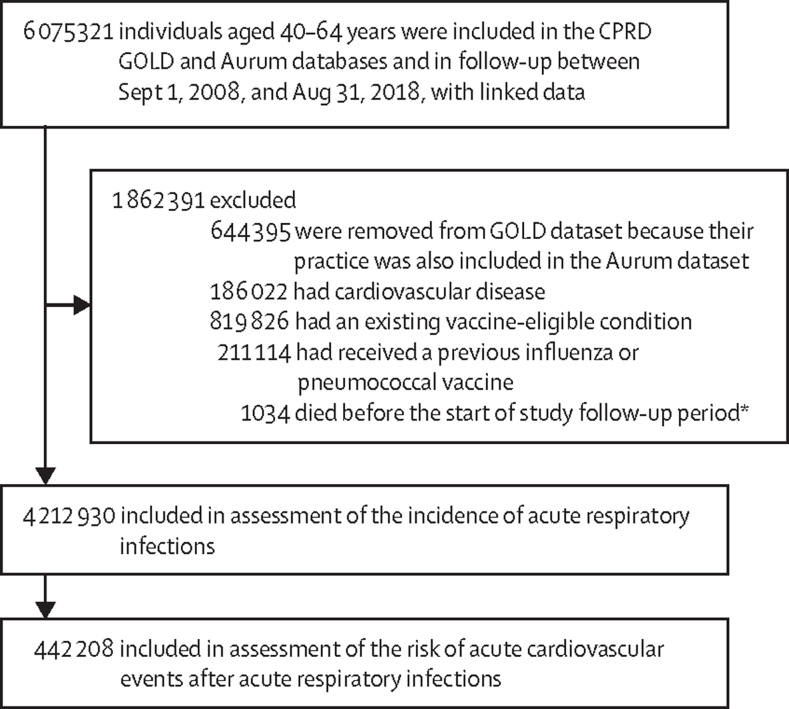
Study profile CPRD=Clinical Practice Research Datalink. *Identified from linked Office for National Statistics mortality data.

6 075 321 individuals aged 40–64 years included in the GOLD and Aurum databases were in follow-up and had linked data in the HES APC between Sept 1, 2008, and Aug 31, 2018, of whom 4 212 930 eligible individuals (773 362 from the GOLD and 3 439 568 from the Aurum database) were included in the final cohort (figure 1). Individuals were followed up for a median of 3·9 years (IQR 1·6–7·6), 2 226 561 (52·9%) of 4 212 898 with available data on sex were men, 1 986 337 (47·1%) were women, and the median age was 46 years (IQR 40–53; table 1). Demographic and lifestyle characteristics of individuals in the GOLD and Aurum datasets were similar, except that higher proportions of individuals in the Aurum dataset were non-White (399 150 [13·0%] of 3 060 297 *vs* 61 461 [9·6%] of 642 421), residing in more deprived regions (627 333 [18·3%] of 3 434 620 *vs* 105 045 [13·6%] of 772 985), and current or former smokers (2 002 856 [60·1%] of 3 334 866 *vs* 393 016 [52·6%] of 747 925) than in the GOLD dataset (appendix p 5).

**Table 1:** Baseline demographic and lifestyle characteristics

	**Cohort (n=4 212 930)**
**Age, years**	
40–44	1 920 369 (45·6%)
45–49	782 897 (18·6%)
50–54	612 202 (14·5%)
55–59	490 619 (11·6%)
60–64	406 843 (9·7%)
**Sex**	
Male	2 226 561/4 212 898 (52·9%)
Female	1 986 337/4 212 898 (47·1%)
**Race or ethnicity**	
White	3 242 107/3 702 718 (87·6%)
South Asian	194 931/3 702 718 (5·3%)
Black	154 270/3 702 718 (4·2%)
Mixed or other	111 410/3 702 718 (3·0%)
**Townsend quintile**	
1 (least deprived)	1 004 670/4 207 605 (23·9%)
2	905 691/4 207 605 (21·5%)
3	825 679/4 207 605 (19·6%)
4	739 187/4 207 605 (17·6%)
5 (most deprived)	732 378/4 207 605 (17·4%)
BMI category*†	
Underweight	51 002/3 447 604 (1·5%)
Normal weight	1 449 683/3 447 604 (42·0%)
Overweight	1 274 701/3 447 604 (37·0%)
Obese	672 218/3 447 604 (19·5%)
Smoking status*	
Non-smoker	1 686 919/4 082 791 (41·3%)
Current smoker	1 076 707/4 082 791 (26·4%)
Former smoker	1 319 165/4 082 791 (32·3%)
Alcohol consumption*	
No known heavy drinking	3 477 336/3 674 494 (94·6%)
Heavy drinking‡	197 158/3 674 494 (5·4%)

Data are n (%) or n/N (%). BMI=body-mass index.

* Closest measure before the start of follow-up.

† Underweight was defined as a BMI of <18·5 kg/m^2^, normal weight as a BMI of 18·5–24·9 kg/m^2^, overweight as a BMI of 25·0–29·9 kg/m^2^, and obese as a BMI of 30·0–39·9 kg/m^2^.

‡ Defined as either a recorded intake of more than 42 units per week or a diagnostic code suggestive of alcohol addiction or excessive alcohol consumption.

526 480 (12·5%) of 4 212 930 individuals had diagnosed hypertension: 347 418 at baseline and a further 179 062 during follow-up. 607 087 (14·4%) individuals had a QRISK2 score of 10% or higher: 402 594 at baseline, and a further 204 493 had a score that increased to 10% or higher during follow-up. 239 184 (5·7%) individuals had both hypertension and a QRISK score of 10% or higher.

586 147 episodes of acute respiratory infection were recorded among 442 408 individuals: 107 639 episodes of influenza or influenza-like illness and 31 068 episodes of pneumonia. The incidence of all-cause acute respiratory infection and pneumonia increased with age, with the exception of the incidence of acute respiratory infection among those with a QRISK2 score of 10% or higher, for which there was no age trend, and the incidence of influenza or influenza-like illness decreased with age (appendix p 18). The incidence of acute respiratory infection was higher among individuals with hypertension (40·3 infections per 1000 person-years [95% CI 40·0–40·7]) and a QRISK2 score of 10% or higher (43·8 infections per 1000 person-years [43·4–44·2]) than among those without hypertension (29·1 infections per 1000 person-years [29·0–29·2]) and a QRISK2 score of less than 10% (28·8 infections per 1000 person-years [28·7–28·9]; table 2).

**Table 2:** Association between cardiovascular risk and acute respiratory infection

	**Number of events**	**Incidence per 1000 person-years (95% CI)**	**Crude IRR (95% CI)**	**Age and sex-adjusted IRR (95% CI)**	Fully adjusted*IRR (95% CI)
**Acute respiratory infection**					
Hypertension	77 674	40·3 (40·0–40·7)	1·38 (1·36–1·39)	1·33 (1·32–1·34)	1·04 (1·03–1·05)
No hypertension	508 473	29·1 (29·0–29·2)	1 (ref)	1 (ref)	1 (ref)
QRISK2 ≥10%	81 662	43·8 (43·4–44·2)	1·52 (1·50–1·53)	NA	1·39 (1·37–1·40)
QRISK2 <10%	504 485	28·8 (28·7–28·9)	1 (ref)	NA	1 (ref)
**Influenza or influenza-like illness**					
Hypertension	12 050	6·3 (6·1–6·4)	1·14 (1·11–1·16)	1·25 (1·22–1·27)	0·98 (0·96–1·00)
No hypertension	95 589	5·5 (5·4–5·5)	1 (ref)	1 (ref)	1 (ref)
QRISK2 ≥10%	10 010	5·4 (5·3–5·5)	0·96 (0·94–0·98)	NA	0·88 (0·86–0·90)
QRISK2 <10%	97 629	5·6 (5·5–5·6)	1 (ref)	NA	1 (ref)
**Pneumonia**					
Hypertension	4479	2·3 (2·2–2·4)	1·59 (1·53–1·65)	1·32 (1·27–1·38)	1·12 (1·07–1·16)
No hypertension	26 589	1·5 (1·5–1·5)	1 (ref)	1 (ref)	1 (ref)
QRISK2 ≥10%	6476	3·5 (3·4–3·6)	2·60 (2·52–2·69)	NA	2·32 (2·25–2·40)
QRISK2 <10%	24 592	1·4 (1·4–1·4)	1 (ref)	NA	1 (ref)

Total person-years per 1000 years of follow-up available was 1926·2 for hypertension, 17 467·9 for no hypertension, 1865·1 for a QRISK2 score of 10% or higher, and 17 529·1 for a QRISK2 score of less than 10%. Likelihood ratio test p values for all comparisons were less than 0·0001. IRR=incidence rate ratio. NA=not applicable.

* Hypertension models were adjusted for age, sex, race or ethnicity, socioeconomic status, body-mass index, alcohol consumption, smoking status, and consultation frequency; and QRISK2 models were adjusted for alcohol consumption and consultation frequency.

In the unadjusted analysis, there was an increased incidence of acute respiratory infection among individuals with hypertension compared with those without hypertension (IRR 1·38 [95% CI 1·36–1·39]) and similarly for individuals with a QRISK2 score of 10% or higher compared with those with a QRISK2 score of less than 10% (1·52 [1·50–1·53]). After adjustment for confounders, the association between hypertension and acute respiratory infection was substantially reduced (IRR 1·04 [95% CI 1·03–1·05]), but the association between a QRISK2 score of 10% or higher and acute respiratory infection remained (1·39 [1·37–1·40]; table 2). The increased incidence of pneumonia among individuals with hypertension compared with those without hypertension (1·12 [1·07–1·16]), and among individuals with a QRISK2 score of 10% or higher compared with those with a QRISK2 score of less than 10% (2·32 [2·25–2·40]) was more pronounced than for all-cause acute respiratory infection (table 2). For influenza and influenza-like illness, the incidence rate ratio was lower among individuals with hypertension than among those without hypertension (0·98 [0·96–1·00]), and lower among individuals with a QRISK2 score of 10% or higher than among those with a QRISK2 score of less than 10% (0·88 [0·86–0·90]; table 2).

The sensitivity analyses, in which we analysed CPRD GOLD and Aurum datasets separately (appendix p 6), redefined the study population (appendix p 7), and compared recorded and calculated QRISK2 scores (appendix p 8), showed similar results to those of the primary analysis.

Among the 442 408 individuals (with 526 800 acute respiratory infection episodes) who had an acute respiratory infection, 4169 (0·9%) had an acute cardiovascular event within 1 year. 1606 (38·5%) of these individuals had pneumonia (appendix p 10).

985 (11·2 events per 1000 person-years [95% CI 10·5–12·0]) acute cardiovascular events occurred in individuals with hypertension, 3184 (5·8 events per 1000 person-years [5·6–6·0]) in those without hypertension, 1526 (17·5 events per 1000 person-years [16·7–18·5]) in individuals with a QRISK2 score of 10% or higher, and 2643 (4·8 events per 1000 person-years [4·6–5·0]) in those with a QRISK2 score of less than 10% (table 3). The number of acute cardiovascular events that occurred in individuals after influenza or influenza-like illness and pneumonia, according to cardiovascular risk, is shown in the appendix (pp 9–10).

**Table 3:** Incidence and risk of acute cardiovascular events after acute respiratory infection by cardiovascular risk group

	**Number of events**	**Incidence per 1000 person-years (95% CI)**	**Crude HR (95% CI)**	**Age and sex-adjusted HR (95% CI)**	Fully adjusted*HR (95% CI)
**Any cardiovascular event**					
Hypertension	985	11·2 (10·5–12·0)	2·08 (1·93–2·23)	1·97 (1·84–2·12)	1·98 (1·83–2·15)
No hypertension	3184	5·8 (5·6–6·0)	1 (ref)	1 (ref)	1 (ref)
QRISK2 ≥10%	1526	17·5 (16·7–18·5)	3·74 (3·51–3·98)	NA	3·65 (3·42–3·89)
QRISK2 <10%	2643	4·8 (4·6–5·0)	1 (ref)	NA	1 (ref)
**Acute coronary syndrome**†					
Hypertension	372	4·2 (3·8–4·7)	2·19 (1·95–2·46)	2·06 (1·83–2·32)	2·13 (1·86–2·44)
No hypertension	1140	2·1 (1·9–2·2)	1 (ref)	1 (ref)	1 (ref)
QRISK2 ≥10%	613	7·0 (6·5–7·6)	4·42 (3·98–4·89)	NA	4·37 (3·93–4·86)
QRISK2 <10%	899	1·6 (1·5–1·7)	1 (ref)	NA	1 (ref)
**Heart failure**					
Hypertension	290	3·3 (2·9–3·7)	2·04 (1·79–2·32)	1·92 (1·69–2·19)	2·08 (1·79–2·42)
No hypertension	961	1·7 (1·6–1·9)	1 (ref)	1 (ref)	1 (ref)
QRISK2 ≥10%	478	5·5 (5·0–6·0)	4·00 (3·57–4·49)	NA	3·85 (3·42–4·34)
QRISK2 <10%	773	1·4 (1·3–1·5)	1 (ref)	NA	1 (ref)
**Acute limb ischaemia**					
Hypertension	25	0·3 (0·2–0·4)	2·98 (1·85–4·78)	2·82 (1·74–4·55)	4·63 (2·68–7·99)
No hypertension	55	0·1 (0·1–0·1)	1 (ref)	1 (ref)	1 (ref)
QRISK2 ≥10%	42	0·5 (0·4–0·7)	7·55 (4·62–11·07)	NA	6·93 (4·43–10·83)
QRISK2 <10%	38	0·1 (0·1–0·1)	1 (ref)	NA	1 (ref)
**Stroke or transient ischaemic attack**‡					
Hypertension	360	4·1 (3·7–4·6)	2·15 (1·91–2·42)	2·08 (1·84–2·34)	2·01 (1·75–2·29)
No hypertension	1120	2·0 (1·9–2·1)	1 (ref)	1 (ref)	1 (ref)
QRISK2 ≥10%	468	5·4 (4·9–5·9)	2·99 (2·68–3·34)	NA	2·93 (2·62–3·28)
QRISK2 <10%	1012	1·8 (1·7–1·9)	1 (ref)	NA	1 (ref)
**Cardiovascular-related death**					
Hypertension	129	1·5 (1·2–1·8)	2·11 (1·73–2·58)	1·99 (1·63–2·43)	2·15 (1·69–2·73)
No hypertension	413	0·7 (0·7–0·8)	1 (ref)	1 (ref)	1 (ref)
QRISK2 ≥10%	230	2·6 (2·3–3·0)	4·77 (4·03–5·66)	NA	4·81 (3·99–5·81)
QRISK2 <10%	312	0·6 (0·5–0·6)	1 (ref)	NA	1 (ref)

Total person-years per 1000 years of follow-up available was 87·9 for hypertension, 553·6 for no hypertension, 87·0 for a QRISK2 score of 10% or higher, and 554·4 for a QRISK2 score of less than 10%. Likelihood ratio test p values for all comparisons were less than 0·0001. HR=hazard ratio. NA=not applicable.

* Hypertension models were adjusted for age, sex, race or ethnicity, socioeconomic status, body-mass index, alcohol consumption, and smoking status; QRISK2 models were adjusted for alcohol consumption.

† Fully adjusted HR for myocardial infarction alone was 2·22 (95% CI 1·91–2·59) in the hypertension model and 4·89 (4·35–5·51) in the QRISK2 model; fully adjusted HR for angina alone was 2·03 (1·51–2·72) in the hypertension model and 3·06 (2·38–3·93) in the QRISK2 model.

‡ Fully adjusted HR for stroke was 2·10 (95% CI 1·81–2·43) in the hypertension model and 2·90 (2·56–3·30) in the QRISK2 model; and fully adjusted HR for transient ischaemic stroke alone was 1·77 (1·34–2·33) in the hypertension model and 3·10 (2·46–3·90) in the QRISK2 model.

2106 (50·5%) of the 4169 acute cardiovascular events occurred within 30 days of an acute respiratory infection (appendix p 19), but the median time interval was slightly longer in individuals with hypertension (median 41 days [IQR 1–168]) or a QRISK2 score of 10% or higher (31 days [1–60]) compared with those without hypertension (24 days [1–156]) or a QRISK2 score of less than 10% (26 days [1–159]). The time interval between pneumonia and an acute cardiovascular event was shorter than for all-cause acute respiratory infection, whereas the time interval for influenza or influenza-like illness was longer (appendix p 19).

After adjustment for confounders, hypertension (hazard ratio [HR] 1·98 [95% CI 1·83–2·15]) and a QRISK2 score of 10% or higher (3·65 [3·42–3·89]) remained associated with acute cardiovascular events after acute respiratory infection (table 3). When acute cardiovascular events were analysed separately, the HRs were largest for acute limb ischaemia, even though there were only a small number of events (hypertension *vs* no hypertension HR 4·63 [95% CI 2·68–7·99]; QRISK2 score of ≥10% *vs* <10% 6·93 [4·43–10·83]), acute coronary syndrome (2·13 [1·86–2·44]; and 4·37 [3·93–4·86]), and cardiovascular death (2·15 [1·69–2·73]; and 4·81 [3·99–5·81]).

**Figure 2:**
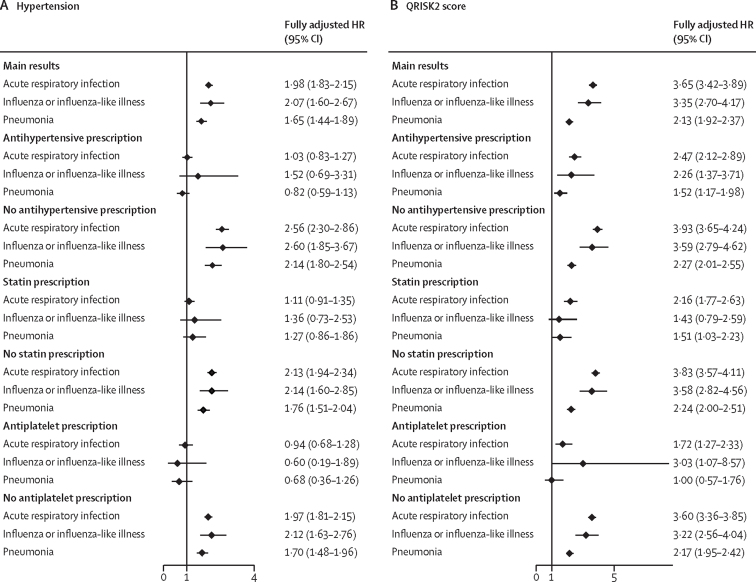
Risk of an acute cardiovascular event after an acute respiratory infection in the hypertension model (A) and in the QRISK2 score model (B) by stratifying factor HRs were adjusted for age, sex, race or ethnicity, socioeconomic status, body-mass index, alcohol intake, and smoking status in the hypertension model, and for alcohol intake in the QRISK2 score model. HR=hazard ratio.

In the analysis stratified by prescription of antihypertensive, statin, or antiplatelet drugs, there was no association between hypertension status and acute cardiovascular events after acute respiratory infection among individuals with a prescription (antihypertensives HR 1·00 [95% CI 0·84–1·18], statins 1·09 [0·91–1·30], antiplatelets 0·86 [0·65–1·13]), but the association remained for those without a prescription (no antihypertensives 2·62 [2·37–2·89], no statins 2·14 [1·98–2·32], no antiplatelets 2·05 [1·91–2·21]). A QRISK2 score of 10% or higher remained associated with acute cardiovascular events after acute respiratory infection in patients with and without a prescription of antihypertensives, statins, or antiplatelets, but was higher among individuals without a prescription (antihypertensives 2·45 [2·11–2·85] *vs* no antihypertensives 4·04 [3·76–4·34], statins 2·22 [1·83–2·69] *vs* no statins 3·93 [3·67–4·21], antiplatelets 1·82 [1·36–2·44] *vs* no antiplatelets 3·68 [3·45–3·93]; figure 2; appendix p 11). The interactions between cardiovascular risk and prescription of antihypertensive, statin and antiplatelet drugs were significant (likelihood ratio test p<0·0001).

The sensitivity analyses, in which we analysed the CPRD GOLD and Aurum datasets separately (appendix pp 12–13), redefined the study population (appendix p 14), compared recorded and calculated QRISK2 scores (appendix p 15), redefined the outcome to include only major adverse cardiovascular events (appendix p 16), and excluded individuals who received influenza or pneumococcal vaccines during the follow-up period (appendix p 17), showed similar results to those of the primary analysis.

## Discussion

In our study population of more than 4·2 million adults aged 40–64 years who did not have chronic health conditions and were ineligible for influenza vaccination, the incidence of both acute respiratory infection and acute cardiovascular events after acute respiratory infection was increased in individuals with elevated cardiovascular risk, measured by diagnosed hypertension or a QRISK2 score of 10% or higher. The incidence of acute respiratory infection was 1·4–1·5 times higher among individuals with increased cardiovascular risk than in those without. After adjustment for confounders, this increase in incidence was marginal when risk was defined by diagnosed hypertension and more substantial when defined by QRISK2 scores. We observed a substantially larger increase in risk of an acute cardiovascular event after respiratory infection when individuals were stratified by QRISK2 score (3·7 times) than by diagnosed hypertension (2·0 times). Associations were similar for all-cause acute respiratory infection, influenza-like illness, and pneumonia. Half of the acute cardiovascular events occurred within 30 days of acute respiratory infection.

Our finding that the incidence of acute respiratory infection was elevated among individuals with increased cardiovascular risk is consistent with the results of a recent UK Biobank study, which examined the association between blood pressure and the risk of different respiratory infections.^14^ Participants with prevalent hypertension had an increased risk of pneumonia (HR 1·36 [95% CI 1·29–1·43]), influenza or viral pneumonia (1·12 [1·01–1·23]), and other lower respiratory infections (1·15 [1·11–1·19]). In our study, the increased incidence of acute respiratory infection among individuals with hypertension, measured by recorded diagnoses, was smaller than observed in the UK Biobank study. Instead, we found more substantial increases in the incidence of acute respiratory infection in individuals with a QRISK2 score of 10% or higher, which includes systolic blood pressure reading and use of antihypertensive drugs. Only 39% (239 194 of 607 087) of individuals with a QRISK2 score of 10% or higher had diagnosed hypertension. Other UK cohort studies have reported increases in the incidence of influenza-like illness to be associated with non-White ethnicity and social deprivation,^22^, ^23^ both of which are cardiovascular risk factors included in the QRISK2 algorithm and can result in higher QRISK2 scores assigned to individuals with these characteristics.

Previous studies have reported transient increases in the risk of myocardial infarction and stroke after acute respiratory infection.^1^, ^2^, ^3^, ^4^ In the few published observational studies that consider the effect of cardiovascular risk on this association, the results were mixed. In two studies, hypertension was associated with an increase in cardiovascular complications after pneumonia.^15^, ^16^ In another study, there was no difference in the likelihood of first myocardial infarction or stroke occurring after acute respiratory infection when stratified by the presence of cardiovascular risk factors.^17^ Studies done during the COVID-19 pandemic have generally shown a high prevalence of cardiovascular disease risk factors in people hospitalised or those who died due to COVID-19. Pooled results suggest that the risk of severe COVID-19 outcomes is 2–3 times higher among hospitalised individuals with hypertension or diabetes than those without hypertension or diabetes.^24^ The degree to which age drives these associations, especially for hypertension, is unclear, with age-adjusted analyses showing varying results.^24^

Several mechanisms could explain the associations between cardiovascular risk factors, acute respiratory infection, and cardiovascular disease. Hypertension could promote immune dysregulation, leading to infection, or the endothelial dysfunction caused by hypertension, hyperlipidaemia, and diabetes could promote infection.^25^, ^26^ Infectious agents, such as SARS-CoV-2, the influenza virus, and *S pneumoniae*, could also exacerbate atherosclerotic processes. The infectious agent could have direct effects on vascular cells, or the infection could induce haemodynamic, inflammatory, and procoagulant processes. The release of proinflammatory cytokines in response to an infection can mediate atherosclerosis or directly affect plaque rupture.^26^ Endothelial dysfunction, caused by a range of cardiovascular risk factors, is a key early stage of atherosclerosis.^26^

A strength of our study is that we used large, population-based, linked datasets generalisable to the UK population. We also compared results across two measures of cardiovascular risk. The marked increase in the incidence of acute cardiovascular events after acute respiratory infection in individuals with a QRISK2 score of 10% of higher compared with those with diagnosed hypertension is consistent with the multiple cardiovascular risk factors accounted for in the QRISK2 score.

Our selected study population should have reduced confounding. We included only individuals without chronic health conditions, who were not thought to be at high risk of acute respiratory infection or complications related to acute respiratory infection, and who were not recommended for influenza vaccination in England, according to current guidance. However, the selective study population prevented comparison of the incidence of acute respiratory infection and acute cardiovascular events after acute respiratory infection among individuals with established cardiovascular disease.

In England, diagnoses of acute respiratory infection are based primarily on clinical judgement, with most cases not laboratory confirmed. We included only codes that were most likely to be representative of systemic infection, which could plausibly induce atherosclerotic processes. However, clinically diagnosed influenza is poorly defined.^27^ An under-estimation of the incidence of some acute respiratory infections, particularly influenza-like illness, is likely to occur because it is a short-lived illness. Conversely, due to its severity, pneumonia will usually result in health-care attendance. These differences in presentation and EHR data capture could account for the differing incidence of influenza-like illness and pneumonia among individuals with increased cardiovascular risk. From 1995 to 2013, recording of influenza-like illness decreased while recording of cough or fever symptoms increased in UK primary care,^27^ and recording of community-acquired pneumonia increased in primary and secondary care.^28^ Recording of symptoms, rather than diagnosis, in individuals with a QRISK2 score of 10% or higher, who were likely to attend primary care for comorbid conditions, could account for the lower incidence of influenza-like illness in these individuals than in those who had a QRISK2 score of less than 10%.

We might have misclassified QRISK2 score due to missing data for variables used in its calculation. However, the similar results from primary care recorded and captured in CPRD and our calculated QRISK2 scores suggest minimal misclassification. Furthermore, we adjusted for consultation frequency, as individuals who infrequently attend primary care services are unlikely to have biometric measures such as BMI or blood pressure recorded. We used a pragmatic definition for hypertension based on coded diagnoses only; this coded diagnosis corresponds to how, if eligible, individuals would be identified for influenza and pneumococcal vaccination in primary care. We are likely to have captured both controlled and uncontrolled hypertension. In the stratified analysis, 38% (25 843 of 68 731) of individuals with diagnosed hypertension had not been prescribed antihypertensive drugs in the year before the acute respiratory infection. Conversely, only 3% (14 912 of 458 069) of individuals without a diagnosis of hypertension were prescribed antihypertensive drugs. Possible misclassification could have biased the results towards the null, which could explain the small association observed between hypertension and acute respiratory infection compared with the larger association for QRISK2 scores in our study and blood pressure reading-defined hypertension in the UK Biobank study.^14^

We assumed that acute cardiovascular events occurring after acute respiratory infection were due to infection. However, some events could have been unrelated, particularly those that occurred several months after infection. The possibility of unrelated events is consistent with our finding of a longer median time between acute respiratory infection and acute cardiovascular events in individuals with increased cardiovascular risk compared with those without an increased risk, as those with an increased cardiovascular risk are more likely to have a cardiovascular event regardless of acute respiratory infection.

Our findings emphasise the importance of improved cardiovascular risk management, which could reduce the incidence of acute respiratory infection and its cardiovascular consequences. The COVID-19 pandemic has highlighted this need. Preventive cardiovascular treatments, particularly among individuals with a QRISK2 score of 10% or higher, could reduce acute respiratory infection-related complications. A meta-analysis of nine observational studies showed a 41% reduction in the odds of mortality within 30 days of having pneumonia among statin users.^29^ Targeted interventions for individuals with acute respiratory infection and a QRISK2 score of 10% or higher should be considered. A CPRD-based study published in 2020 found that aspirin use after hospitalisation for pneumonia resulted in a 54% reduction in the incidence of myocardial infarction and a 30% reduction in the incidence of stroke.^30^

Typically, individuals with increased cardiovascular risk do not receive seasonal influenza or pneumococcal vaccines. Previous RCTs have shown that the influenza vaccine provides secondary prevention of cardiovascular disease.^7^ However, no RCTs or observational studies have investigated the use of vaccines for primary prevention of cardiovascular complications. Extending influenza vaccination to individuals with a QRISK2 score of 10% or higher would, on the basis of the most recent year in our dataset (2017–18), include approximately 150 000 individuals in England. Vaccinating these individuals could reduce cardiovascular complications and presumably decrease the incidence of influenza and associated complications, such as hospitalisation and mortality. Future studies using laboratory data to ascertain whether specific organisms lead to increases in cardiovascular events in people with increased cardiovascular risk would inform potential expansion of vaccine prioritisation.

In conclusion, the results of our study suggest that the incidence of acute respiratory infection, particularly pneumonia, is increased in people with high cardiovascular risk, and that the incidence of acute cardiovascular events after acute respiratory infection is also increased in these individuals. The QRISK2 score provides a better measure of the risk of a first cardiovascular event following acute respiratory infection than a diagnosis of hypertension alone. Therefore, the QRISK2 score could be used not only to identify individuals who require cardiovascular risk management, but also to target prevention and treatment of acute respiratory infection to individuals at increased risk of cardiovascular events following infection.

## Data sharing

The data used for this study were obtained from the CPRD. All CPRD data are available via an application to the Independent Scientific Advisory Committee (see https://www.cprd.com/Data-access). Data acquisition is associated with a fee and data protection requirements. This study is supported by code lists used to define each health condition, which have been made openly available at https://doi.org/10.17037/DATA.00002240. Our data management and analysis computer code is available via GitHub at https://github.com/jenAdavidson/cvrisk_mace_ari. All code is shared without investigator support.

## Declaration of interests

AB reports receiving grants from AstraZeneca, UK Research and Innovation, and the National Institute for Health Research (NIHR). CW-G reports receiving grants from the Wellcome Trust and the British Heart Foundation; receiving speaker fees from Sanofi Pasteur; and participating in a data safety monitoring board for an investigator-led trial of the effect of influenza vaccination after heart attack on future cardiovascular prognosis (NCT02831608) from January, 2019, to April, 2020. HIM is funded by the NIHR Health Protection Research Unit in Immunisation. JAD is funded by the British Heart Foundation through the grant received by CW-G. All other authors declare no competing interests.
