# Supplementary material for: Risk of acute respiratory infection and acute cardiovascular events following acute respiratory infection among adults with increased cardiovascular risk in England between 2008 and 2018: a retrospective, population-based cohort study
Source: Lancet Digit Health. 2021 Nov 22;3(12):e773–83. doi: 10.1016/S2589-7500(21)00203-X (PMC8628002; doi:10.1016/S2589-7500(21)00203-X)
Supplement: Supplementary appendix [file mmc1.pdf]

THE LANCET
Digital Health

Supplementary appendix

This appendix formed part of the original submission and has been peer reviewed. We post it as supplied by the authors.

Supplement to: Davidson JA, Banerjee A, Smeeth L, et al. Risk of acute respiratory infection and acute cardiovascular events following acute respiratory infection among adults with increased cardiovascular risk in England between 2008 and 2018: a retrospective, population-based cohort study. *Lancet Digit Health* 2021; **3:** e773–83.

Supplementary material

Contents

Supplementary methods

Definitions used for excluded health conditions 2

Study period and follow-up outline........ 3

Acute respiratory infection episode structure........ 3

Calculating QRISK2 scores........ 4

Covariate and effect modifier selection 4

Comparing results from recorded and calculated QRISK2 scores........ 4

Supplementary tables

Supplementary table 1. Baseline demographic and lifestyle characteristics of included study population separated by database........ 5

Supplementary table 2. Database comparison of acute respiratory infection incidence rates and incidence rate ratios 6

Supplementary table 3. Crude and adjusted incidence rate ratios for the association between cardiovascular risk and ARI among sensitivity analysis study population........ 7

Supplementary table 4. Crude and adjusted incidence rate ratios for the association between QRISK2 score and ARI, by QRISK2 score identification method 8

Supplementary table 5. Acute cardiovascular events after influenza/ILI incidence rates and hazard ratios by cardiovascular risk group........ 9

Supplementary table 6. Acute cardiovascular events after pneumonia incidence rates and incidence rate ratios by cardiovascular risk group........ 10

Supplementary table 7. Acute cardiovascular events after ARI by anti-hypertensives, statins and antiplatelets prescription status 11

Supplementary table 8. Database comparison of Acute cardiovascular events after ARI incidence rates and hazard ratios by cardiovascular risk group........ 12

Supplementary table 9. Crude and adjusted hazard ratios for the association between cardiovascular risk and Acute cardiovascular events after ARI among sensitivity analysis study population........ 14

Supplementary table 10. Crude and adjusted hazard ratios for the association between QRISK2 score and Acute cardiovascular events after acute respiratory infection, by QRISK2 score identification method... 15

Supplementary table 11. MACE after infection sensitivity analysis results........ 16

Supplementary table 12. Crude and adjusted incidence rate ratios for the association between cardiovascular risk and M Acute cardiovascular events ACE after ARI among only patients who did not receive influenza or pneumococcal vaccine during follow-up 17

Supplementary figures

Supplementary figure 1. Age-specific infection rates by cardiovascular risk group........ 18

Supplementary figure 2. Timing between acute respiratory infection and major adverse cardiovascular event........ 19

Supplementary material references........ 20

Study protocol 21

Definitions used for excluded health conditions

Health condition	Study definition
Cardiovascular disease (CVD)	Any previous clinical diagnosis, major intervention for, or clinical review specific to CVD including heart disease (congenital or otherwise), heart failure, stroke or transient ischaemic attack.
Chronic liver disease	Any previous clinical diagnosis of, or clinical review specific to, chronic liver disease including cirrhosis, oesophageal varices, biliary atresia and chronic hepatitis.
Chronic kidney disease (CKD)	Any previous clinical diagnosis of, or clinical review specific to, CKD stages 3-5, history of dialysis or renal transplant. Or with estimated glomerular filtration rate to classify CKD stages 3-5.[1] Only stages 4-5 excluded from sensitivity analysis using pneumococcal vaccine recommendations.
Chronic respiratory disease (not asthma)	Any previous clinical diagnosis of, or clinical review specific to, chronic respiratory disease, including chronic obstructive pulmonary disease, emphysema, bronchitis, cystic fibrosis, or fibrosing interstitial lung diseases.
Asthma	Any previous clinical diagnosis of, or clinical review specific to, asthma with at least two prescriptions of inhaled steroids in the year before baseline. Or any previous hospitalisation for asthma. Not excluded from sensitivity analysis using pneumococcal vaccine recommendations.
Chronic neurological disease	Any previous clinical diagnosis of, or clinical review specific to, a neurological disease such as Parkinson's disease, motor neurone disease, multiple sclerosis (MS), cerebral palsy, dementia or a learning/intellectual disability. Not excluded from sensitivity analysis using pneumococcal vaccine recommendations.
Diabetes mellitus	Any previous diagnosis of, or clinical review specific to, diabetes mellitus, or with a prescription for medication used to treat diabetes. Only treated diabetes excluded from sensitivity analysis using pneumococcal vaccine recommendations.
Asplenia/sickle cell disease	Any previous clinical diagnosis of, or clinical review specific to, asplenia or dysfunction of the spleen (including sickle cell disease but not sickle cell trait).
Severe obesity	Latest body mass index before baseline was ≥40 kg/m^2. Not excluded from sensitivity analysis using pneumococcal vaccine recommendations.
Immunosuppression	Any previous clinical diagnosis of, or clinical review specific to, HIV, solid organ transplant or other permanent immunosuppression (such as genetic conditions compromising immune function).
	Previous clinical diagnosis of, or clinical review specific to, aplastic anaemia or haematological malignancy, or receiving a bone marrow or stem cell transplant in the 2 years before baseline.
	Previous clinical diagnosis of, or clinical review specific to, other/unspecified immune deficiency or receiving chemotherapy or radiotherapy in the year before baseline.
	Prescription of biological therapy or at least 2 prescriptions for oral steroids or other immunosuppressants including DMARDS, Methotrexate, Azathioprine, or corticosteroid injections in the year before baseline.

Study period and follow-up outline

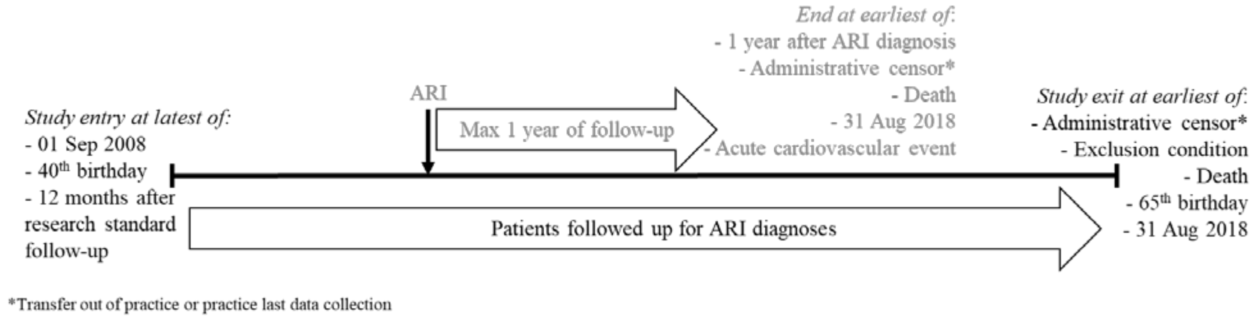

Acute respiratory infection episode structure

Records containing ARI codes were identified in both CPRD and HES. All ARI codes were used to define the primary outcome (ARI) and subsets were used to define the secondary outcomes of influenza/influenza-like-illness (ILI) and pneumonia.

To account for multiple consultations related to one illness, CPRD or HES records dated within 28 days of each other were regarded as part of the same illness-episode. The earliest record in the episode determined the date of illness for the primary outcome of ARI and for the secondary outcome of influenza/ILI. By comparison, for the secondary outcome of pneumonia, the earliest record which was coded as pneumonia determined the date of illness. Figure A below illustrates the episode structure. Influenza/ILI and pneumonia episodes were structured differently to account for the aetiological differences in the conditions. In an episode where there was an influenza/ILI record, but this was not the first record, it is likely that the original presentation was due to influenza/ILI but not identified as such at the time. Conversely, pneumonia can develop and worsen over time from the original infection.

In analysis of ARI outcomes, to account for multiple episodes of illness per patient, an "observation” period ended at the date of ARI and a new “observation” period begin. In analysis of acute cardiovascular events after ARI, an ARI episode triggered a one-year follow-up. Further ARI episodes which occurred in that further up were counted and adjusted for in analysis.

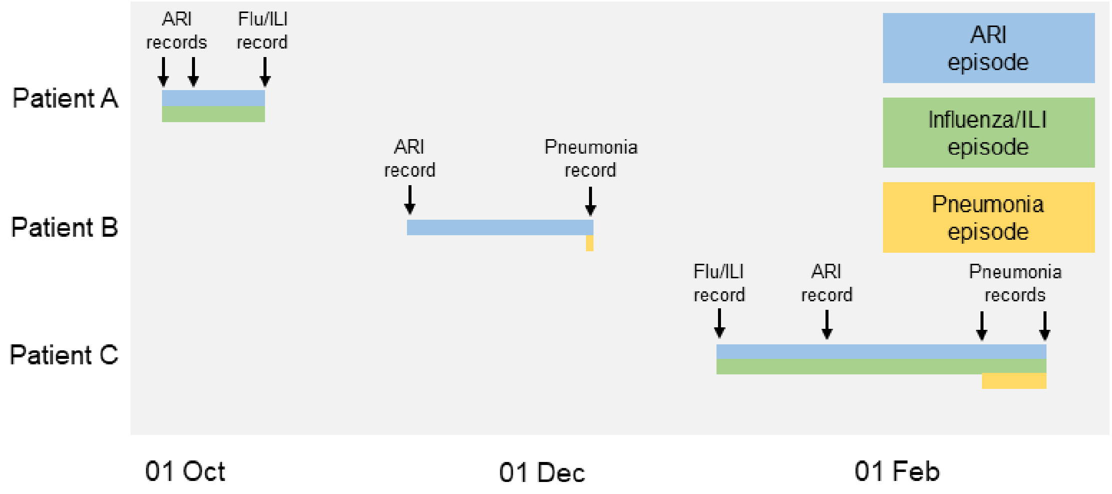

Figure A. Example patient episode classifications

Calculating QRISK2 scores

An individual's QRISK2 score is calculated based on age, sex, ethnicity, deprivation score from linked Townsend data, diabetes, family history of coronary heart disease in a first degree relative <60 years, atrial fibrillation, chronic kidney disease stage 4 or 5, rheumatoid arthritis, ratio of total serum cholesterol to high density lipoprotein cholesterol, systolic blood pressure, treated hypertension, body-mass index, and smoking status.[2] The complete QRISK2 algorithm used to calculate score has never been published but was updated annually during its use from 2008-2017. Using the information which the authors have published online (https://qrisk.org/), the London School of Hygiene and Tropical Medicine Electronic Health Record Research Group wrote Stata program files to calculate scores based on the information published for the 2015 version of QRISK2 and Quality and Outcomes Framework Read and SNOMED codes[3] (with the exception of chronic kidney disease stage 4 or 5 which used all available Read and SNOMED codes). A population-average imputation approach was used to account for missing data, to reflect the QRISK2 algorithm used in clinical practice. Our QRISK2 score process is published at https://zenodo.org/record/3981238.

Of note diabetes, chronic kidney disease and severe obesity are determinants of a higher QRISK2 score, but individuals with these conditions were excluded from our study (existing eligibility for influenza vaccination).

Covariate and effect modifier selection

We adjusted for age and sex as standard. Additionally, older age is associated with increase in cardiovascular risk[4] as well as ARI.[5] Men have higher cardiovascular risk than women,[4] while there is suggestion that women are more likely to experience ARI.[6] Non-White ethnicity is associated with both cardiovascular risk[4] and ARI.[7] Smoking, alcohol intake and BMI are important lifestyle factors, along with socio-economic deprivation, which effect the likelihood of many health conditions. Consultation frequency was adjusted for as hypertension (along with the factors included in the QRISK2 algorithm) and ARI diagnoses are more frequent in patients who regularly attend primary care services. No comorbid health conditions were included due to patients with many possible confounding conditions excluded from our study population.

Antihypertensives, statins and antiplatelets were considered effect modifiers in our analysis of cardiovascular risk and acute cardiovascular events after ARI, with stratified analysis conducted. Patients with diagnosed hypertension are likely to be prescribed antihypertensive treatments. Antihypertensive prescriptions were not used to classify patients with hypertension. Patients prescribed antihypertensives may have better-controlled hypertension and be less likely to experience an acute cardiovascular event following an ARI. Similarly, patients with hypertension may also be prescribed cholesterol-lowering medication. Individuals with a high QRISK2 score, many of whom have hypertension, are offered statin treatment.[8]

Comparing results from recorded and calculated QRISK2 scores

In main analyses, our classification of cardiovascular risk level based on QRISK2 was done using our own algorithm. To validate the results obtained from our algorithm we repeated the main analyses for ARI outcome and acute cardiovascular events after ARI restricted to patients with a QRISK2 score recorded in CPRD directly by GPs in 2015-2017. We chose this time period as our algorithm was based on the 2015 version of QRISK2, and minimal changes were made to QRISK2 from 2015-2017.

In CPRD GOLD we identified patients with QRISK2 scores using Read codes "22W..00" , "38DF.00" or "38DP.00" from the Clinical file. If any of these three codes were recorded for consultations between 2015-2017, then the corresponding results was obtained from the Additional file. In CPRD Aurum, we used SNOMED codes "718087004", "763244005", "1085871000000105", "1656451000006101", "1656461000006104", "810931000000108" from the Observation file.

In our subpopulation of patients with recorded QRISK2 scores we started follow-up from the latest of first recorded QRISK2 on or after 1 January 2015 or start of follow-up date from main analysis. Follow-up ended at the earliest of 31 December 2017 or end of follow-up date from main analysis.

Supplementary table 1. Baseline demographic and lifestyle characteristics of included study population separated by database

	Gold n = 773,362	Aurum n = 3,439,568
Age (years)		
40-44	316,376 (40·9%)	1,603,993 (46·6%)
45-49	152,207 (19·7%)	630,690 (18·3%)
50-54	121,385 (15·7%)	490,817 (14·3%)
55-59	99,557 (12·9%)	391,062 (11·4%)
60-64	83,837 (10·8%)	323,006 (9·4%)
Sex	n = 773,351	n = 3,439,547
Male	405,608 (52·4%)	1,820,953 (52·9%)
Female	367,743 (47·6%)	1,618,594 (47·1%)
Ethnicity	n = 642,421	n = 3,060,297
White	580,960 (90·4%)	2,661,147 (87·0%)
South Asian	24,419 (3·8%)	170,512 (5·6%)
Black	18,343 (2·9%)	135,927 (4·4%)
Mixed/Other	18,699 (2·9%)	92,711 (3·0%)
Townsend quintile	n = 772,985	n = 3,434,620
1 (least deprived)	190,835 (24·7%)	813,835 (23·7%)
2	180,189 (23·3%)	725,502 (21·1%)
3	158,379 (20·5%)	667,300 (19·4%)
4	138,537 (17·9%)	600,650 (17·5%)
5 (most deprived)	105,045 (13·6%)	627,333 (18·3%)
BMI category*	n = 772,985	n = 2,803,225
Underweight (<18·5 kg/m^2)	9,269 (1·4%)	41,733 (1·5%)
Normal weight (18·5-24·9 kg/m^2)	271,221 (42·1%)	1,178,462 (42·0%)
Overweight (25·0-29·9 kg/m^2)	238,478 (37·0%)	1,036,223 (37·0%)
Obese (30·0-39·9 kg/m^2)	125,411 (19·5%)	546,807 (19·5%)
Smoking status*	n = 747,925	n = 3,334,866
Non-smoker	354,909 (47·5%)	1,332,010 (39·9%)
Current smoker	193,384 (25·9%)	883,323 (26·5%)
Ex-smoker	199,632 (26·7%)	1,119,533 (33·6%)
Alcohol consumption*	n = 678,452	n = 2,996,042
Not a heavy drinker	654,536 (96·5%)	2,822,800 (94·2%)
Heavy drinker	26,109 (3·5%)	173,242 (5·8%)

Data are n (%). *Closest measure before start of follow-up.

Supplementary table 2. Database comparison of acute respiratory infection incidence rates and incidence rate ratios

Outcome	Cardiovascular risk	Database	Rate per 1,000 person-years (95% CI)		Crude IRR (95% CI)	Age and sex-adjusted IRR (95% CI)	Fully-adjusted* IRR (95% CI)
			High risk	Low risk			
ARI	Hypertension	GOLD	39·5 (38·7-40·4)	29·9 (29·6-30·1)	1·32 (1·29-1·35)	1·29 (1·26-1·32)	1·08 (1·05-1·11)
		Aurum	40·5 (40·1-40·9)	29·0 (28·9-29·2)	1·39 (1·37-1·40)	1·34 (1·32-1·35)	1·05 (1·03-1·06)
		GOLD and Aurum	40·3 (40·0-40·7)	29·1 (29·0-29·2)	1·38 (1·36-1·39)	1·33 (1·32-1·34)	1·04 (1·03-1·05)
		Meta-analysis of GOLD and Aurum	-	-	1·36 (1·29-1·43)	1·32 (1·27-1·37)	1·06 (1·03-1·09)
	QRISK2 ≥10%	GOLD	43·9 (42·9-44·8)	29·6 (29·3-29·8)	1·49 (1·45-1·52)	-	1·37 (1·34-1·41)
		Aurum	43·8 (43·3-44·2)	28·6 (28·5-28·8)	1·52 (1·51-1·54)	-	1·39 (1·37-1·40)
		GOLD and Aurum	43·8 (43·4-44·2)	28·8 (28·7-28·9)	1·52 (1·50-1·53)	-	1·39 (1·37-1·40)
		Meta-analysis of GOLD and Aurum	-	-	1·51 (1·48-1·54)	-	1·39 (1·37-1·40)
Influenza / ILI	Hypertension	GOLD	6·2 (5·9-6·5)	5·6 (5·5-5·7)	1·10 (1·05-1·16)	1·22 (1·16-1·29)	1·01 (0·96-1·07)
		Aurum	6·3 (6·1-6·4)	5·5 (5·4-5·5)	1·14 (1·12-1·17)	1·25 (1·22-1·28)	0·98 (0·95-1·00)
		GOLD and Aurum	6·3 (6·1-6·4)	5·5 (5·4-5·5)	1·14 (1·11-1·16)	1·25 (1·22-1·27)	0·98 (0·96-1·00)
		Meta-analysis of GOLD and Aurum	-	-	1·13 (1·09-1·16)	1·25 (1·22-1·27)	0·99 (0·96-1·01)
	QRISK2 ≥10%	GOLD	5·1 (4·8-5·3)	5·7 (5·6-5·8)	0·89 (0·84-0·94)	-	0·83 (0·78-0·88)
		Aurum	5·4 (5·3-5·5)	5·5 (5·5-5·6)	0·97 (0·95-0·99)	-	0·89 (0·87-0·91)
		GOLD and Aurum	5·4 (5·3-5·5)	5·6 (5·5-5·6)	0·96 (0·94-0·98)	-	0·88 (0·86-0·90)
		Meta-analysis of GOLD and Aurum	-	-	0·93 (0·86-1·01)	-	0·87 (0·81-0·92)
Pneumonia	Hypertension	GOLD	1·9 (1·7-2·1)	1·3 (1·3-1·4)	1·46 (1·32-1·61)	1·25 (1·13-1·39)	1·07 (0·97-1·19)
		Aurum	2·4 (2·3-2·5)	1·6 (1·5-1·6)	1·61 (1·55-1·68)	1·33 (1·28-1·39)	1·12 (1·07-1·17)
		GOLD and Aurum	2·3 (2·2-2·4)	1·5 (1·5-1·5)	1·59 (1·53-1·65)	1·32 (1·27-1·38)	1·12 (1·07-1·16)
		Meta-analysis of GOLD and Aurum	-	-	1·55 (1·40-1·69)	1·31 (1·25-1·38)	1·11 (1·07-1·16)
	QRISK2 ≥10%	GOLD	3·1 (2·9-3·3)	1·2 (1·2-1·3)	2·62 (2·41-2·86)	-	2·37 (2·17-2·60)
		Aurum	3·5 (3·4-3·6)	1·4 (1·4-1·5)	2·59 (2·50-2·69)	-	2·31 (2·23-2·39)
		GOLD and Aurum	3·5 (3·4-3·6)	1·4 (1·4-1·4)	2·60 (2·52-2·69)	-	2·32 (2·25-2·40)
		Meta-analysis of GOLD and Aurum	-	-	2·60 (2·51-2·68)	-	2·32 (2·24-2·39)

In meta-analysis the between database heterogeneity was assessed using the I^2 statistic. I^2 results for fully-adjusted estimates: ARI and hypertension=67%, ARI and QRISK2=6%, influenza/ILI and hypertension=0%, influenza/ILI and QRISK2=79%, pneumonia and hypertension=0%, pneumonia and QRISK2=0%.
*Hypertension models adjusted for: age, sex, ethnicity, socioeconomic status, BMI, alcohol intake, smoking status and consultation frequency. QRISK2 models adjusted for: alcohol intake and consultation frequency.

Supplementary table 3. Crude and adjusted incidence rate ratios for the association between cardiovascular risk and ARI among sensitivity analysis study population

Outcome	Cardiovascular risk	No. of events	Rate per 1,000 person-years (95% CI)	Crude IRR (95% CI)	Age and sex-adjusted IRR (95% CI)	Fully-adjusted* IRR (95% CI)
Acute respiratory infection	Hypertension	96,203	42·7 (42·4-43·1)	1·40 (1·38-1·41)	1·34 (1·33-1·36)	1·04 (1·03-1·05)
	No hypertension	558,302	30·4 (30·3-30·5)	1	1	1
	QRISK2 ≥10%	96,193	45·8 (45·4-46·2)	1·51 (1·50-1·52)	-	1·37 (1·36-1·39)
	QRISK2 <10%	558,312	30·1 (30·0-30·2)	1	-	1
Influenza/ILI	Hypertension	14,436	6·4 (6·3-6·5)	1·14 (1·12-1·17)	1·25 (1·23-1·27)	0·98 (0·96-1·00)
	No hypertension	102,207	5·6 (5·5-5·6)	1	1	1
	QRISK2 ≥10%	11,573	5·5 (5·4-5·6)	0·97 (0·95-0·99)	-	0·88 (0·86-0·90)
	QRISK2 <10%	105,070	5·7 (5·6-5·7)	1	-	1
Pneumonia	Hypertension	5,700	2·5 (2·5-2·6)	1·61 (1·56-1·67)	1·35 (1·30-1·39)	1·11 (1·07-1·16)
	No hypertension	29,987	1·6 (1·6-1·7)	1	1	1
	QRISK2 ≥10%	7,798	3·7 (3·6-3·8)	2·59 (2·51-2·67)	-	2·30 (2·23-2·37)
	QRISK2 <10%	27,889	1·5 (1·5-1·5)	1	-	1

Total person-years per 1,000: hypertension = 2,251·4, no hypertension = 18,373·1, QRISK2 ≥10% = 2,099·5 and QRISK2 <10% = 18,524·0. LRT p-values all <0·0001. *Hypertension models adjusted for: age, sex, ethnicity, socioeconomic status, BMI, alcohol intake, smoking status and consultation frequency. QRISK2 models adjusted for: alcohol intake and consultation frequency.

Supplementary table 4. Crude and adjusted incidence rate ratios for the association between QRISK2 score and ARI, by QRISK2 score identification method

Cardiovascular risk method	No. of events	Rate per 1,000 person-years	Crude IRR (95% CI)	Alcohol intake adjusted IRR (95% CI)
Recorded QRISK2 ≥10%	6,018	27·6 (26·8-28·4)	1·41 (1·37-1·46)	1·42 (1·38-1·47)
Recorded QRISK2 <10%	22,561	19·7 (19·5-20·0)	1	1
Calculated QRISK2 ≥10%	4,296	29·6 (28·6-30·6)	1·48 (1·43-1·54)	1·40 (1·35-1·46)
Calculated QRISK2 <10%	24,283	20·0 (19·7-20·3)	1	1

Total person-years per 1,000: calculated QRISK2 ≥10% = 145·30, calculated QRISK2 <10% = 1,215·49, recorded QRISK2 ≥10% = 218·09 and recorded QRISK2 <10% = 1,142·70.

Supplementary table 5. Acute cardiovascular events after influenza/ILI incidence rates and hazard ratios by cardiovascular risk group

Outcome	Cardiovascular risk	No. of events	Rate per 1,000 person-years (95% CI)	Crude HR (95% CI)	Age and sex-adjusted HR (95% CI)	Fully-adjusted* HR (95% CI)
Any event	Hypertension	99	8·0 (6·6-9·8)	2·24 (1·79-2·80)	2·13 (1·70-2·66)	2·07 (1·60-2·67)
	No hypertension	352	3·7 (3·3-4·1)	1	1	1
	QRISK2 ≥10%	116	11·5 (9·7-13·9)	3·36 (2·72-4·15)	-	3·35 (2·70-4·17)
	QRISK2 <10%	335	3·4 (3·1-3·8)	1	-	1
ACS	Hypertension	34	2·8 (2·0-3·9)	1·99 (1·36-2·90)	1·87 (1·28-2·73)	1·97 (1·30-2·99)
	No hypertension	136	1·4 (1·2-1·7)	1	1	1
	QRISK2 ≥10%	51	5·1 (3·9-6·8)	4·15 (2·99-5·77)	-	4·04 (2·89-5·63)
	QRISK2 <10%	119	1·2 (1·0-1·5)	1	-	1
Heart failure	Hypertension	21	1·7 (1·1-2·7)	2·85 (1·73-4·69)	2·69 (1·63-4·43)	2·82 (1·53-5·21)
	No hypertension	59	0·6 (0·5-0·8)	1	1	1
	QRISK2 ≥10%	22	2·2 (1·5-3·4)	3·68 (2·25-6·01)	-	3·72 (2·23-6·19)
	QRISK2 <10%	58	0·6 (0·5-0·8)	1	-	1
Stroke or TIA	Hypertension	45	3·6 (2·7-5·0)	2·33 (1·67-3·25)	2·24 (1·60-3·13)	2·04 (1·39-2·98)
	No hypertension	153	1·6 (1·4-1·9)	1	1	1
	QRISK2 ≥10%	41	4·1 (3·0-5·6)	2·53 (1·80-3·57)	-	2·49 (1·74-3·57)
	QRISK2 <10%	157	1·6 (1·4-1·9)	1	-	1
CVD death	Hypertension	12	1·0 (0·6-1·8)	2·81 (1·46-5·41)	2·66 (1·38-5·12)	3·31 (1·51-7·25)
	No hypertension	34	0·4 (0·3-0·5)	1	1	1
	QRISK2 ≥10%	13	1·3 (0·8-2·4)	3·82 (2·01-7·26)	-	4·19 (2·17-8·07)
	QRISK2 <10%	33	0·3 (0·2-0·5)	1	-	1

Acute limb ischaemia not included as secondary outcome due to event numbers <10. Total person-years per 1,000: hypertension = 12·3, no hypertension = 95·7, QRISK2 ≥10% = 10·0 and QRISK2 <10% = 98·0. LRT p-values all <0·0001. *Hypertension models adjusted for: age, sex, ethnicity, socioeconomic status, BMI, alcohol intake and smoking status. QRISK2 models adjusted for: alcohol intake. †Results subdivided into MI and unstable angina separately: MI fully-adjusted HR in hypertension model 2·34 (95% CI 1·48-3·71) and in QRISK2 model 4·84 (95% CI 3·36-6·97), angina fully-adjusted HR in hypertension model 0·89 (95% CI 0·30-2·61) and in QRISK2 model 2·17 (95% CI 0·96-4·91). ‡Results subdivided into stroke and TIA separately: stroke fully-adjusted HR in hypertension model 2·40 (95% CI 1·54-3·74) and in QRISK2 model 2·69 (95% CI 1·76-4·12), TIA fully-adjusted HR in hypertension model 1·49 (95% CI 0·76-2·91) and in QRISK2 model 2·47 (95% CI 1·33-4·56).

Supplementary table 6. Acute cardiovascular events after pneumonia incidence rates and incidence rate ratios by cardiovascular risk group

Outcome	Cardiovascular risk	No. of events	Rate per 1,000 person-years (95% CI)	Crude HR (95% CI)	Age and sex-adjusted HR (95% CI)	Fully-adjusted* HR (95% CI)
Any event	Hypertension	341	105·6 (94·5-118·3)	1·62 (1·44-1·82)	1·58 (1·41-1·78)	1·65 (1·44-1·89)
	No hypertension	1,265	63·6 (60·1-67·4)	1	1	1
	QRISK2 ≥10%	574	130·9 (120·1-142·9)	2·17 (1·96-2·40)	-	2·13 (1·92-2·37)
	QRISK2 <10%	1,032	55·1 (51·8-58·7)	1	-	1
ACS	Hypertension	117	36·1 (30·1-43·6)	1·73 (1·41-2·12)	1·68 (1·37-2·06)	1·91 (1·50-2·44)
	No hypertension	405	20·3 (18·4-22·5)	1	1	1
	QRISK2 ≥10%	217	49·5 (43·3-56·9)	2·75 (2·31-3·26)	-	2·82 (2·35-3·38)
	QRISK2 <10%	305	16·2 (14·5-18·2)	1	-	1
Heart failure	Hypertension	145	44·8 (38·1-53·1)	1·59 (1·32-1·90)	1·56 (1·30-1·87)	1·70 (1·37-2·11)
	No hypertension	545	27·4 (25·2-29·8)	1	1	1
	QRISK2 ≥10%	253	57·7 (50·9-65·9)	2·26 (1·94-2·64)	-	2·20 (1·87-2·58)
	QRISK2 <10%	437	23·3 (21·2-25·7)	1	-	1
Acute limb ischaemia	Hypertension	10	3·1 (1·7-6·3)	2·32 (1·12-4·80)	2·30 (1·11-4·76)	3·19 (1·34-7·55)
	No hypertension	25	1·3 (0·9-1·9)	1	1	1
	QRISK2 ≥10%	15	3·4 (2·1-6·0)	3·21 (1·65-6·26)	-	3·26 (1·65-6·42)
	QRISK2 <10%	20	1·1 (0·7-1·7)	1	-	1
Stroke or TIA	Hypertension	124	38·4 (32·2-46·1)	1·85 (1·52-2·26)	1·83 (1·50-2·23)	1·79 (1·43-2·24)
	No hypertension	400	20·1 (18·2-22·2)	1	1	1
	QRISK2 ≥10%	160	36·5 (31·2-42·9)	1·71 (1·42-2·06)	-	1·67 (1·38-2·04)
	QRISK2 <10%	364	19·4 (17·5-21·6)	1	-	1
CVD death	Hypertension	58	17·9 (13·9-23·5)	1·60 (1·20-2·14)	1·56 (1·17-2·08)	1·57 (1·11-2·23)
	No hypertension	219	11·0 (9·6-12·6)	1	1	1
	QRISK2 ≥10%	120	27·4 (22·9-33·0)	3·02 (2·39-3·83)	-	3·11 (2·39-4·04)
	QRISK2 <10%	157	8·4 (7·2-9·8)	1	-	1

Total person-years per 1,000: hypertension = 3·2, no hypertension = 19·9, QRISK2 ≥10% = 4·4 and QRISK2 <10% = 18·7. LRT p-values all <0·0001. *Hypertension models adjusted for: age, sex, ethnicity, socioeconomic status, BMI, alcohol intake and smoking status. QRISK2 models adjusted for: alcohol intake. †Results subdivided into MI and unstable angina separately: MI fully-adjusted HR in hypertension model 1·94 (95% CI 1·48-2·54) and in QRISK2 model 3·04 (95% CI 2·49-3·70), angina fully-adjusted HR in hypertension model 2·42 (95% CI 1·38-4·22) and in QRISK2 model 2·03 (95% CI 1·24-3·33). ‡Results subdivided into stroke and TIA separately: stroke fully-adjusted HR in hypertension model 1·78 (95% CI 1·42-2·23) and in QRISK2 model 1·77 (95% CI 1·45-2·15), TIA fully-adjusted HR in hypertension model 1·74 (95% CI 0·74-4·08) and in QRISK2 model 1·14 (95% CI 0·56-2·34).

Supplementary table 7. Acute cardiovascular events after ARI by anti-hypertensives, statins and antiplatelets prescription status

Prescribed treatment of interest	Cardiovascular risk	No. of events	Total person-years per 1,000	Rate per 1,000 person-years (95% CI)	Crude HR (95% CI)	Age and sex-adjusted HR (95% CI)	Fully-adjusted* HR (95% CI)
Anti-hypertensives (n=57,800)	Hypertension (n=42,888)	532	55·6	9·6 (8·8-10·4)	1·00 (0·84-1·18)	0·86 (0·72-1·02)	1·03 (0·83-1·27)
	No hypertension (n=14,912)	183	19·0	9·6 (8·4-11·2)	1	1	1
	QRISK2 ≥10% (n=22,805)	436	28·9	15·1 (13·7-16·6)	2·45 (2·11-2·85)	-	2·47 (2·12-2·89)
	QRISK2 <10% (n=34,995)	279	45·6	6·1 (5·4-6·9)	1	-	1
No anti-hypertensives (n=469,000)	Hypertension (n=25,843)	453	32·2	14·1 (12·8-15·4)	2·62 (2·37-2·89)	2·58 (2·34-2·85)	2·56 (2·30-2·86)
	No hypertension (n=443,157)	3,001	534·5	5·6 (5·4-5·8)	1	1	1
	QRISK2 ≥10% (n=49,332)	1,090	58·1	18·8 (17·7-19·9)	4·04 (3·76-4·34)	-	3·93 (3·65-4·24)
	QRISK2 <10% (n=419,668)	2,364	508·7	4·6 (4·5-4·8)	1	-	1
Statins (n=33,837)	Hypertension (n=16,865)	246	21·9	11·3 (9·9-12·8)	1·09 (0·91-1·30)	1·06 (0·88-1·26)	1·11 (0·91-1·35)
	No hypertension (n=16,972)	228	22·1	10·3 (9·1-11·8)	1	1	1
	QRISK2 ≥10% (n=16,746)	322	21·5	15·0 (13·4-16·7)	2·22 (1·83-2·69)	-	2·16 (1·77-2·63)
	QRISK2 <10% (n=17,091)	152	22·5	6·8 (5·8-8·0)	1	-	1
No statins (n=492,963)	Hypertension (n=51,866)	739	66·0	11·2 (10·4-12·1)	2·14 (1·98-2·32)	2·09 (1·93-2·27)	2·13 (1·94-2·34)
	No hypertension (n=441,097)	2,956	531·4	5·6 (5·4-5·8)	1	1	1
	QRISK2 ≥10% (n=55,391)	1,204	65·5	18·4 (17·4-19·5)	3·93 (3·67-4·21)	-	3·83 (3·57-4·11)
	QRISK2 <10% (n=437,572)	2,491	531·8	4·7 (4·5-4·9)	1	-	1
Antiplatelets (n=7,670)	Hypertension (n=3,658)	88	4·4	20·0 (16·3-24·9)	0·86 (0·65-1·13)	0·78 (0·59-1·04)	0·94 (0·68-1·28)
	No hypertension (n=4,012)	112	4·9	22·9 (19·1-27·8)	1	1	1
	QRISK2 ≥10% (n=3,970)	131	4·7	28·2 (23·7-33·7)	1·82 (1·36-2·44)	-	1·72 (1·27-2·33)
	QRISK2 <10% (n=3,700)	69	4·6	14·9 (11·8-19·1)	1	-	1
No antiplatelets (n=519,130)	Hypertension (n=65,073)	897	83·4	10·8 (10·1-11·5)	2·05 (1·91-2·21)	1·96 (1·82-2·11)	1·97 (1·81-2·15)
	No hypertension (n=454,057)	3,072	548·6	5·6 (54-5·8)	1	1	1
	QRISK2 ≥10% (n=68,167)	1,395	82·4	16·9 (16·1-17·9)	3·68 (3·45-3·93)	-	3·60 (3·36-3·85)
	QRISK2 <10% (n=450,963)	2,574	549·7	4·7 (4·5-4·9)	1	-	1

*Hypertension models adjusted for: age, sex, ethnicity, socioeconomic status, BMI, alcohol intake and smoking status. QRISK2 models adjusted for: alcohol intake.

Supplementary table 8. Database comparison of acute cardiovascular events after ARI incidence rates and hazard ratios by cardiovascular risk group

Outcome	Cardiovascular risk	Database	Rate per 1,000 person-years (95% CI)		Crude HR (95% CI)	Age and sex-adjusted HR (95% CI)	Fully-adjusted* HR (95% CI)
			High risk	Low risk			
Any event	Hypertension	GOLD	11·5 (9·7-13·6)	5·9 (5·4-6·4)	2·03 (1·68-2·46)	1·92 (1·59-2·33)	2·06 (1·67-2·54)
		Aurum	11·2 (10·4-12·0)	5·7 (5·5-6·0)	2·08 (1·93-2·25)	1·98 (1·84-2·14)	1·97 (1·80-2·16)
		GOLD and Aurum	11·2 (10·5-12·0)	5·8 (5·5-6·0)	2·08 (1·93-2·23)	1·97 (1·84-2·12)	1·98 (1·83-2·15)
		Meta-analysis of GOLD and Aurum	-	-	2·07 (1·93-2·22)	1·97 (1·83-2·11)	1·98 (1·82-2·15)
	QRISK2	GOLD	17·2 (15·0-19·9)	5·1 (4·6-5·6)	3·37 (2·85-4·00)	-	3·35 (2·81-3·99)
		Aurum	17·6 (16·7-18·6)	4·7 (4·5-4·9)	3·80 (3·55-4·07)	-	3·70 (3·44-3·97)
		GOLD and Aurum	17·5 (16·7-18·5)	4·8 (4·6-5·0)	3·65 (3·42-3·89)	-	3·65 (3·42-3·89)
		Meta-analysis of GOLD and Aurum	-	-	3·67 (3·27-4·06)	-	3·63 (3·35-3·91)
ACS	Hypertension	GOLD	4·8 (3·7-6·2)	2·2 (1·9-2·6)	2·24 (1·66-3·02)	2·10 (1·55-2·83)	2·18 (1·54-3·08)
		Aurum	4·1 (3·7-4·6)	2·0 (1·9-2·2)	2·18 (1·92-2·48)	2·06 (1·81-2·34)	2·12 (1·83-2·46)
		GOLD and Aurum	4·2 (3·8-4·7)	2·1 (1·9-2·2)	2·19 (1·95-2·46)	2·06 (1·83-2·32)	2·13 (1·86-2·44)
		Meta-analysis of GOLD and Aurum	-	-	2·19 (1·93-2·45)	2·07 (1·82-2·31)	2·13 (1·84-2·42)
	QRISK2	GOLD	7·0 (5·7-8·8)	1·9 (1·6-2·2)	3·66 (2·80-4·79)	-	3·79 (2·87-5·00)
		Aurum	7·0 (6·5-7·7)	1·6 (1·5-1·7)	4·56 (4·08-5·10)	-	4·48 (3·99-5·03)
		GOLD and Aurum	7·0 (6·5-7·6)	1·6 (1·5-1·7)	4·42 (3·98-4·89)	-	4·37 (3·93-4·86)
		Meta-analysis of GOLD and Aurum	-	-	4·22 (3·36-5·01)	-	4·30 (3·70-4·89)
Heart failure	Hypertension	GOLD	4·2 (3·2-5·6)	1·7 (1·5-2·0)	2·53 (1·83-3·49)	2·39 (1·74-3·30)	2·83 (1·96-4·08)
		Aurum	3·2 (2·8-3·6)	1·7 (1·6-1·9)	1·96 (1·69-2·26)	1·85 (1·60-2·13)	1·96 (1·65-2·32)
		GOLD and Aurum	3·3 (2·9-3·7)	1·7 (1·6-1·9)	2·04 (1·79-2·32)	1·92 (1·69-2·19)	2·08 (1·79-2·42)
		Meta-analysis of GOLD and Aurum	-	-	2·11 (1·62-2·59)	1·99 (1·53-2·45)	2·24 (1·44-3·04)
	QRISK2	GOLD	5·5 (4·3-7·1)	1·6 (1·3-1·9)	3·50 (2·58-4·73)	-	3·43 (2·51-4·70)
		Aurum	5·5 (5·0-6·1)	1·4 (1·3-1·5)	4·10 (3·62-4·63)	-	3·93 (3·45-4·48)
		GOLD and Aurum	5·5 (5·0-6·0)	1·4 (1·3-1·5)	4·00 (3·57-4·49)	-	3·85 (3·42-4·34)
		Meta-analysis of GOLD and Aurum	-	-	3·99 (3·53-4·45)	-	3·84 (3·37-4·31)
Acute limb ischaemia	Hypertension	GOLD	0·0 (0·0-0·0)	0·1 (0·1-0·2)	-	-	-
		Aurum	0·3 (0·2-0·5)	0·1 (0·1-0·1)	3·49 (2·15-5·68)	3·32 (2·03-5·43)	5·72 (3·25-10·08)
		GOLD and Aurum	0·3 (0·2-0·4)	0·1 (0·1-0·1)	2·98 (1·85-4·78)	2·82 (1·74-4·55)	4·63 (2·68-7·99)
		Meta-analysis of GOLD and Aurum	-	-	-	-	-
	QRISK2	GOLD	0·3 (0·1-1·3)	0·1 (0·0-0·2)	4·18 (0·99-17·68)	-	4·21 (0·99-17·85)

Outcome	Cardiovascular risk	Database	Rate per 1,000 person-years (95% CI)		Crude HR (95% CI)	Age and sex-adjusted HR (95% CI)	Fully-adjusted* HR (95% CI)
			High risk	Low risk			
		Aurum	0·5 (0·4-0·7)	0·1 (0·0-0·1)	7·56 (4·76-12·00)	-	7·34 (4·56-11·80)
		GOLD and Aurum	0·5 (0·4-0·7)	0·1 (0·1-0·1)	7·15 (4·62-11·07)	-	6·93 (4·43-10·83)
		Meta-analysis of GOLD and Aurum	-	-	7·03 (3·70-10·35)	-	6·85 (3·53-10·18)
Stroke or TIA	Hypertension	GOLD	4·0 (3·1-5·4)	2·0 (1·7-2·4)	2·06 (1·50-2·85)	1·96 (1·42-2·71)	2·12 (1·50-2·99)
		Aurum	4·1 (3·7-4·6)	2·0 (1·9-2·2)	2·17 (1·91-2·46)	2·10 (1·84-2·38)	1·99 (1·72-2·30)
		GOLD and Aurum	4·1 (3·7-4·6)	2·0 (1·9-2·1)	2·15 (1·91-2·42)	2·08 (1·84-2·34)	2·01 (1·75-2·29)
		Meta-analysis of GOLD and Aurum	-	-	2·15 (1·90-2·41)	2·08 (1·83-2·33)	2·01 (1·74-2·28)
	QRISK2	GOLD	5·6 (4·4-7·2)	1·8 (1·5-2·1)	3·08 (2·29-4·14)	-	2·91 (2·14-3·95)
		Aurum	5·3 (4·9-5·9)	1·8 (1·7-2·0)	2·98 (2·65-3·35)	-	2·93 (2·59-3·31)
		GOLD and Aurum	5·4 (4·9-5·9)	1·8 (1·7-1·9)	2·99 (2·68-3·34)	-	2·93 (2·62-3·28)
		Meta-analysis of GOLD and Aurum	-	-	2·99 (2·67-3·32)	-	2·93 (2·59-3·26)
CVD death	Hypertension	GOLD	1·4 (0·9-2·3)	0·7 (0·5-0·9)	2·06 (1·19-3·56)	1·96 (1·13-3·40)	2·31 (1·13-4·71)
		Aurum	1·5 (1·2-1·8)	0·8 (0·7-0·8)	2·12 (1·72-2·63)	2·00 (1·62-2·47)	2·13 (1·65-2·74)
		GOLD and Aurum	1·5 (1·2-1·8)	0·7 (0·7-0·8)	2·11 (1·73-2·58)	1·99 (1·63-2·43)	2·15 (1·69-2·73)
		Meta-analysis of GOLD and Aurum	-	-	2·11 (1·69-2·54)	2·00 (1·60-2·39)	2·15 (1·62-2·67)
	QRISK2	GOLD	2·5 (1·8-3·7)	0·5 (0·4-0·7)	4·58 (2·87-7·31)	-	4·86 (2·91-8·11)
		Aurum	2·7 (2·3-3·1)	0·6 (0·5-0·6)	4·80 (4·00-5·76)	-	4·80 (3·92-5·87)
		GOLD and Aurum	2·6 (2·3-3·0)	0·6 (0·5-0·6)	4·77 (4·03-5·66)	-	4·81 (3·99-5·81)
		Meta-analysis of GOLD and Aurum	-	-	4·77 (3·95-5·58)	-	4·81 (3·89-5·72)

In meta-analysis the between database heterogeneity was assessed using the I^2 statistic. The I^2 for all pooled estimates was 0%, with the exception of; QRISK2 and acute cardiovascular events (crude I^2=44%, fully-adjusted I^2=11%), QRISK2 and ACS (crude I^2=60%, fully-adjusted I^2=23%), and hypertension and heart failure (crude and age- and sex-adjusted I^2=38%, fully-adjusted I^2=58%). *Hypertension models adjusted for: age, sex, ethnicity, socioeconomic status, BMI, alcohol intake and smoking status. QRISK2 models adjusted for: alcohol intake.

Supplementary table 9. Crude and adjusted hazard ratios for the association between cardiovascular risk and acute cardiovascular events after ARI among sensitivity analysis study population

Outcome	Cardiovascular risk	No. of events	Rate per 1,000 person-years (95% CI)	Crude HR (95% CI)	Age and sex-adjusted HR (95% CI)	Fully-adjusted* HR (95% CI)
Any event	Hypertension	1,263	11·6 (11·0-12·3)	2·12 (1·98-2·26)	2·04 (1·91-2·18)	2·04 (1·89-2·19)
	No hypertension	3,560	5·9 (5·7-6·1)	1	1	1
	QRISK2 ≥10%	1,820	17·8 (17·0-18·6)	3·70 (3·49-3·92)	-	3·61 (3·40-3·84)
	QRISK2 <10%	3,003	4·9 (4·7-5·1)	1	-	1
ACS	Hypertension	437	4·0 (3·7-4·4)	2·10 (1·88-2·34)	2·01 (1·80-2·24)	2·08 (1·83-2·36)
	No hypertension	1,240	2·0 (1·9-2·2)	1	1	1
	QRISK2 ≥10%	695	6·8 (6·3-7·3)	4·31 (3·92-4·75)	-	4·29 (3·88-4·74)
	QRISK2 <10%	982	1·6 (1·5-1·7)	1	-	1
Heart failure	Hypertension	392	3·6 (3·3-4·0)	2·14 (1·91-2·40)	2·06 (1·83-2·31)	2·16 (1·89-2·47)
	No hypertension	1,098	1·8 (1·7-1·9)	1	1	1
	QRISK2 ≥10%	592	5·8 (5·3-6·3)	4·02 (3·62-4·46)	-	3·90 (3·49-4·34)
	QRISK2 <10%	898	1·5 (1·4-1·6)	1	-	1
Acute limb ischaemia	Hypertension	30	0·3 (0·2-0·4)	3·13 (2·01-4·89)	3·00 (1·92-4·70)	5·01 (2·99-8·38)
	No hypertension	56	0·1 (0·1-0·1)	1	1	1
	QRISK2 ≥10%	47	0·5 (0·3-0·6)	7·34 (4·80-11·20)	-	7·17 (4·65-11·07)
	QRISK2 <10%	39	0·1 (0·0-0·1)	1	-	1
Stroke or TIA	Hypertension	450	4·1 (3·8-4·5)	2·14 (1·92-2·38)	2·09 (1·87-2·33)	2·04 (1·80-2·30)
	No hypertension	1,250	2·1 (1·9-2·2)	1	1	1
	QRISK2 ≥10%	555	5·4 (5·0-5·9)	2·95 (2·67-3·27)	-	2·90 (2·61-3·22)
	QRISK2 <10%	1,145	1·9 (1·8-2·0)	1	-	1
CVD death	Hypertension	173	1·6 (1·4-1·9)	2·20 (1·85-2·62)	2·12 (1·78-2·52)	2·23 (1·81-2·75)
	No hypertension	472	0·8 (0·7-0·9)	1	1	1
	QRISK2 ≥10%	270	2·6 (2·3-3·0)	4·39 (3·76-5·14)	-	4·37 (3·68-5·18)
	QRISK2 <10%	375	0·6 (0·6-0·7)	1	-	1

Total person-years per 1,000: hypertension = 108·6, no hypertension = 606·8, QRISK2 ≥10% = 102·4 and QRISK2 <10% = 613·0. LRT p-values all <0·0001.
*Hypertension models adjusted for: age, sex, ethnicity, socioeconomic status, BMI, alcohol intake and smoking status. QRISK2 models adjusted for: alcohol intake.

Supplementary table 10. Crude and adjusted hazard ratios for the association between QRISK2 score and acute cardiovascular events after acute respiratory infection, by QRISK2 score identification method

Cardiovascular risk method	No. of events	Rate per 1,000 person-years	Crude HR (95% CI)	Alcohol intake adjusted HR (95% CI)
Recorded QRISK2 ≥10%	110	22·6 (18·8-27·3)	3·31 (2·57-4·26)	3·34 (2·58-4·32)
Recorded QRISK2 <10%	132	6·8 (5·7-8·1)	1	1
Calculated QRISK2 ≥10%	89	25·3 (20·6-31·2)	3·43 (2·65-4·46)	3·39 (2·60-4·42)
Calculated QRISK2 <10%	153	7·4 (6·3-8·6)	1	1

Total person-years per 1,000: calculated QRISK2 ≥10% = 3·51, calculated QRISK2 <10% = 20·75, recorded QRISK2 ≥10% = 4·87 and recorded QRISK2 <10% = 19·40.

Supplementary table 11. MACE after infection sensitivity analysis results

Infection type	Cardiovascular risk	No. of events	Rate per 1,000 person-years (95% CI)	Crude HR (95% CI)	Age and sex-adjusted HR (95% CI)	Fully-adjusted* HR (95% CI)
ARI	Hypertension	850	9·7 (9·0-10·4)	2·11 (1·95-2·28)	2·00 (1·85-2·16)	2·02 (1·85-2·21)
	No hypertension	2,711	4·9 (4·7-5·1)	1	1	1
	QRISK2 ≥10%	1,321	15·2 (14·4-16·0)	3·82 (3·57-4·09)	-	3·71 (3·46-3·99)
	QRISK2 <10%	2,240	4·0 (3·9-4·2)	1	-	1
Influenza/ILI	Hypertension	84	6·8 (5·5-8·5)	2·50 (1·96-3·19)	2·36 (1·85-3·03)	2·37 (1·79-3·15)
	No hypertension	268	2·8 (2·5-3·2)	1	1	1
	QRISK2 ≥10%	96	9·6 (7·9-11·8)	3·64 (2·88-4·60)	-	3·59 (2·82-4·59)
	QRISK2 <10%	256	2·6 (2·3-3·0)	1	-	1
Pneumonia	Hypertension	326	100·9 (90·2-113·3)	1·65 (1·47-1·87)	1·62 (1·43-1·83)	1·65 (1·44-1·90)
	No hypertension	1,182	59·5 (56·1-63·1)	1	1	1
	QRISK2 ≥10%	544	124·1 (113·6-135·7)	2·20 (1·98-2·44)	-	2·15 (1·93-2·40)
	QRISK2 <10%	964	51·5 (48·3-55·0)	1	-	1

*Hypertension models adjusted for: age, sex, ethnicity, socioeconomic status, BMI, alcohol intake and smoking status. QRISK2 models adjusted for: alcohol intake.

Supplementary table 12. Crude and adjusted incidence rate ratios for the association between cardiovascular risk and acute cardiovascular events after ARI among only patients who did not receive influenza or pneumococcal vaccine during follow-up

Cardiovascular risk	No. of events	Rate per 1,000 person-years (95% CI)	Crude HR (95% CI)	Age and sex-adjusted HR (95% CI)	Fully-adjusted* HR (95% CI)
Hypertension	985	12·5 (11·8-13·3)	2·17 (2·02-2·33)	2·06 (1·92-2·22)	2·07 (1·90-2·24)
No hypertension	3,184	6·2 (5·9-6·4)	1	1	1
QRISK2 ≥10%	1,526	20·2 (19·2-21·3)	4·06 (3·81-4·32)	-	3·96 (3·70-4·22)
QRISK2 <10%	2,643	5·1 (4·9-5·3)	1	-	1

Patients from 35,505 ARI episodes received influenza or pneumococcal vaccine. Among patients with raised cardiovascular risk, a higher proportion were vaccinated (hypertension=10%, 6,898/68,731 and QRISK2 ≥10%=13%, 9,330/72,137) compared with low cardiovascular risk (no hypertension=6%, 28,607/458,069 and QRISK2 <10%=6%, 26,175/454,663). None of the vaccinated patients had an acute cardiovascular event during follow-up. *Hypertension models adjusted for: age, sex, ethnicity, socioeconomic status, BMI, alcohol intake and smoking status. QRISK2 models adjusted for: alcohol intake.

Supplementary figure 1. Age-specific infection rates by cardiovascular risk group

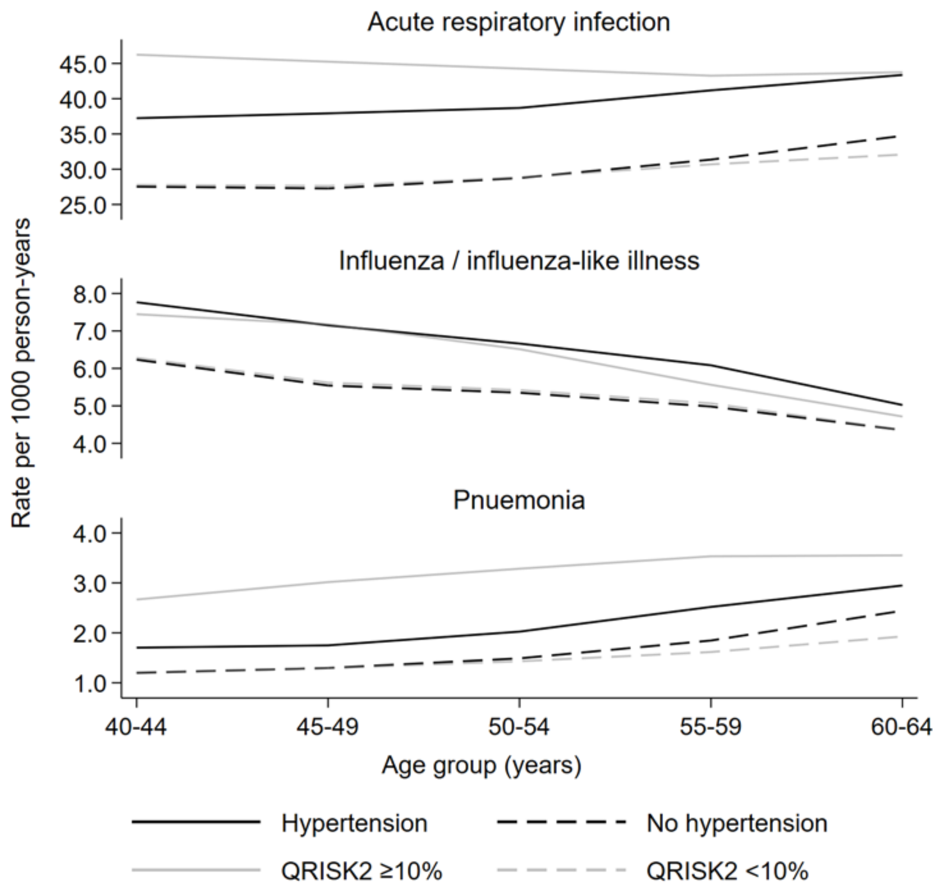

Supplementary figure 2. Timing between acute respiratory infection and major adverse cardiovascular event

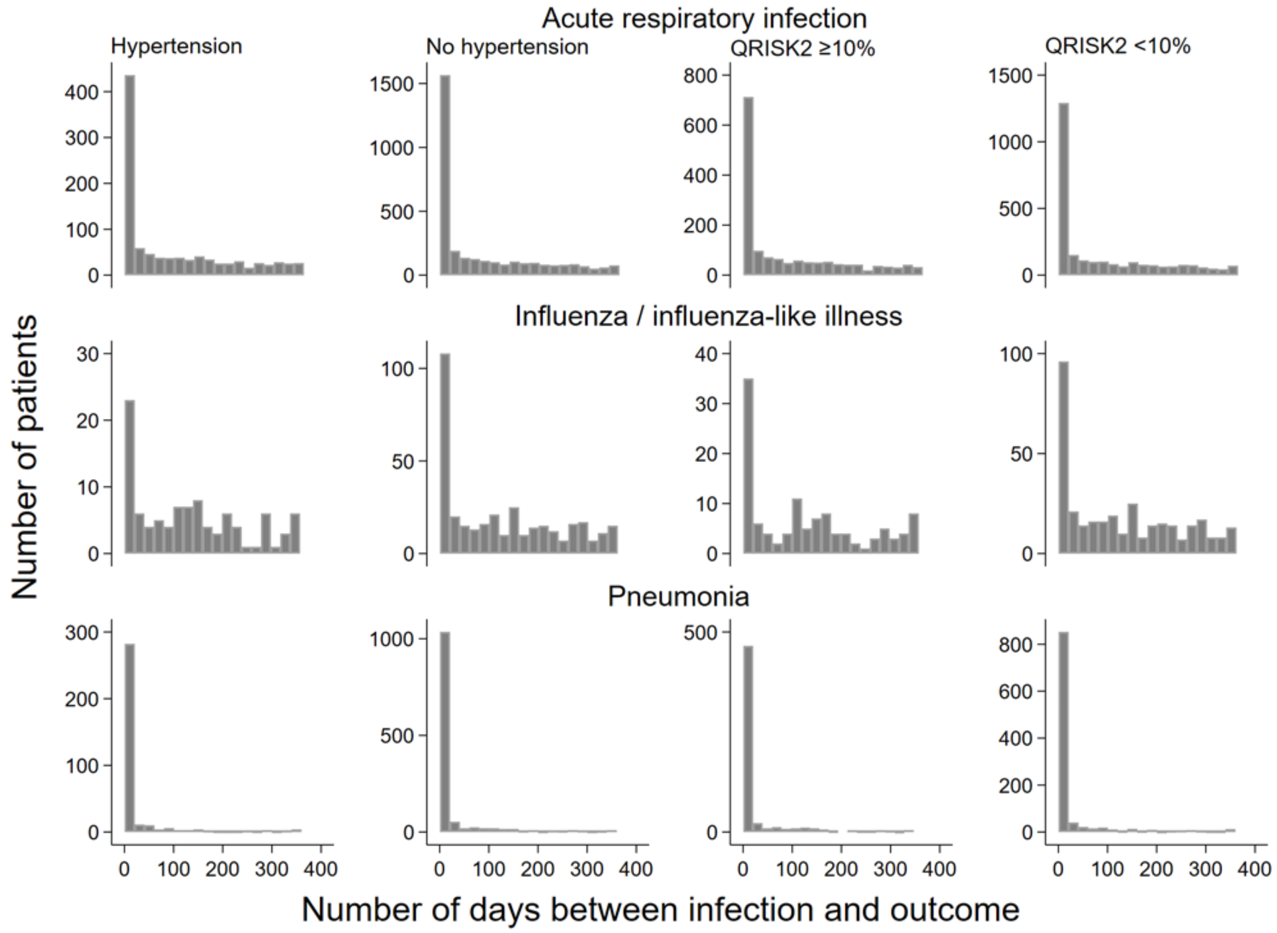

Supplementary material references

1. Levey AS, Stevens LA, Schmid CH, et al. A new equation to estimate glomerular filtration rate. *Ann Intern Med*. 2009;150(9):604-612. doi:10.7326/0003-4819-150-9-200905050-00006

2. Hippisley-Cox J, Coupland C, Vinogradova Y, et al. Predicting cardiovascular risk in England and Wales: prospective derivation and validation of QRISK2. *BMJ*. 2008;336(7659):1475-1482. doi:10.1136/bmj.39609.449676.25

3. National Health Service. Quality and Outcomes Framework (QOF). https://digital.nhs.uk/data-and-information/data-collections-and-data-sets/data-collections/quality-and-outcomes-framework-qof. Published 2020. Accessed March 3, 2020.

4. Damen JAAG, Hooft L, Schuit E, et al. Prediction models for cardiovascular disease risk in the general population: systematic review. *BMJ*. 2016;353:i2416. doi:10.1136/bmj.i2416

5. Talbot HK. Influenza in Older Adults. *Infect Dis Clin North Am*. 2017;31(4):757-766. doi:10.1016/j.idc.2017.07.005

6. Klein SL, Hodgson A, Robinson DP. Mechanisms of sex disparities in influenza pathogenesis. *J Leukoc Biol*. 2012;92(1):67-73. doi:10.1189/jlb.0811427

7. Davidson J, Banerjee A, Mathur R, et al. Ethnic differences in the incidence of clinically diagnosed influenza: an England population-based cohort study 2008-2018. *Wellcome Open Res*. 2021;6:49. doi:10.12688/wellcomeopenres.16620.3

8. National Institute for Health and Care Excellence. *CVD Risk Assessment and Management*.; 2019. https://cks.nice.org.uk/cvd-risk-assessment-and-management. Accessed August 14, 2019.

Study protocol

Applicants must complete all sections listed below<br>Sections which do not apply should be completed as '*Not Applicable*' and justification provided
**A. Study Title (Max. 255 characters)**<br>Effect of raised cardiovascular risk on the rates of acute respiratory infections and subsequent cardiovascular complications: a cohort study using electronic health records
**B. Lay Summary (Max. 250 words)**<br>Heart disease, stroke and lower respiratory tract infections are among the global leading causes of ill-health and death. In 2017, cardiovascular disease (CVD) accounted for more than 160,000 deaths in the UK. Previous research has shown that people have a short-term risk of heart attack and stroke in the days after a serious respiratory infection, such as flu or pneumonia. This risk has mostly been found in older adults and those with pre-existing CVD.<br><br>This study aims to establish whether this risk also occurs in people without pre-existing CVD but who have raised cardiovascular risk, for example high blood pressure, which is suggestive of future CVD. Establishing such risk is important for UK vaccine policy; influenza and pneumococcal vaccination recommendations currently include people aged ≥65 years and those with CVD, but not people aged <65 years with raised cardiovascular risk.<br><br>We will use routinely collected healthcare data in England to compare the occurrence of serious respiratory infections as well as subsequent cardiovascular events, such as heart attack or stroke, in people with a raised cardiovascular risk compared to people without raised risk.<br><br>The results from this study will inform future research to evaluate the impact of influenza and pneumococcal vaccination in populations with raised cardiovascular risk.
**C. Technical Summary (Max. 300 words)**<br>In the UK there are an estimated 7 million people living with CVD, for which the annual costs are estimated to be £19 billion. As the population ages and multimorbidity prevalence increases, stratified interventions are ever more important. The risk of cardiovascular complications after an acute systemic respiratory infection in people with raised cardiovascular risk but without established CVD is unknown. Quantifying any such increased risk will inform whether these groups should be considered for influenza and pneumococcal vaccination.<br><br>Our cohort study will use CPRD data linked to HES and ONS mortality data to increase ascertainment of respiratory and cardiovascular events. We will define cardiovascular risk by hypertension diagnosis and QRISK2 score. QRISK2 is a prediction algorithm for future CVD which utilises a range of risk factors, beyond hypertension, to determine risk. We will first calculate age-specific incidence rates for diagnosis of acute systemic respiratory infections by cardiovascular risk among adults aged 40 to 64 years. We will compare 1) people with hypertension to those without hypertension and 2) people with a QRISK2 score ≥10% in ten years compared to those with a QRISK2 score <10%. We will then use Poisson regression models with Lexis expansion by age group and cardiovascular risk level to estimate incidence rates and rate ratios for 1) acute systemic respiratory infections including influenza-like illness and pneumonia, and 2) major acute cardiovascular events (MACE). Using Cox proportional hazards regression multivariable models, which adjust for potential confounders, we will then estimate the effect of cardiovascular risk on MACE after an acute systemic respiratory infection. In our definition of MACE we will include; myocardial infarction, unstable angina, left ventricular heart failure, stroke, transient ischaemic attack, acute limb ischaemia and cardiovascular death.
**D. Outcomes to be Measured**<br>***Primary***<br>Aim 1 & 2: all-cause acute systemic respiratory tract infections. This includes clinical or confirmed diagnoses such as pneumonia, acute bronchitis, influenza / influenza-like illness (ILI), and other acute infections suggestive of lower respiratory tract improvement.

Aim 3 & 4: all-cause major adverse cardiovascular events (MACE). This includes; cardiovascular death, acute coronary syndrome (ACS) which captures both myocardial infarction (MI) and unstable angina, stroke, transient ischaemic attack (TIA), left ventricular heart failure and acute ischaemic limb.

Secondary
Aim 1 & 2:

- Influenza / ILI
- pneumonia

Aim 3 & 4 cause-specific acute cardiovascular events:

- ACS,
- stroke / TIA,
- left ventricular heart failure,
- acute ischaemic limb, and
- cardiovascular death.

E. Objectives, Specific Aims and Rationale

Research objective
We will use CPRD GOLD and Aurum with linked HES and ONS mortality data to investigate whether the occurrence of cardiovascular complications after acute systemic respiratory infections varies by cardiovascular risk level. Cardiovascular risk will be defined in two separate ways; presence or absence of hypertension diagnosis (exposure group A) and 10-year predicted cardiovascular risk based on calculated QRISK2 score of ≥10% compared to <10% (exposure group B).

Specific aims

- To describe annual age-specific rates of medical attendance for acute respiratory infection (ARI) by cardiovascular risk level in exposure group A and B.
- Compare the effect of cardiovascular risk level (in exposure group A and B) on ARI, particularly ILI and pneumonia, rates using multivariable Poisson regression with Lexis expansion models.
- Compare the effect of cardiovascular risk level (in exposure group A and B) on MACE rates using multivariable Poisson regression with Lexis expansion models.
- By cardiovascular risk level (in exposure group A and B) compare the effect of ARI on cardiovascular complications using multivariable Cox regression models.

Rationale
ARIs are known to trigger cardiovascular events among the elderly and people with existing CVD. Influenza and pneumococcal vaccination are already recommended for these groups. Establishing the risk of cardiovascular complications after an ARI in people with hypertension and with a QRISK2 ≥10% in 10 years (who are not currently recommended for vaccination) could lead to the prevention of premature cardiovascular events and inform whether UK influenza and pneumococcal vaccine policy should be extended to include these population groups.

Hypertension is one of the primary risk factors for future CVD, however it is only one element of risk. QRISK2 provides an individualised CVD risk score based on multiple morbidities and risk factors including blood pressure. Some patients will be defined as high cardiovascular risk based on both blood pressure and QRISK2 score, while others will only be captured by one classification. Ideally, all patients with high cardiovascular risk could be identified based on QRISK2 scores recorded in primary care records. Unfortunately, this score is currently only routinely recorded for a small subset of patients. The morbidities and risk factors on which QRISK2 scores are based are generally well recorded in primary care records allowing scores to be calculated in research datasets.

F. Study Background
Globally, ischaemic heart disease (IHD) and stroke, have been the leading causes of death for more than 15 years, accounting for 15.2 million deaths in 2016 (1). More than 13 million people globally were estimated to have suffered a stroke in 2016 (2), resulting in healthcare expenditure associated with stroke between an estimated 3% and 5% (3–5). While CVD mortality rates in the UK have fallen steadily in the last four decades, in 2017 CVD still accounted for 168,472 deaths, at a rate of 246 per 100,000 people (6). Additionally, the prevalence of CVD has remained stable for the past decade; IHD prevalence was 3.5% in

2007/8 and 3.1% in 2017/8, while stroke and TIA prevalence was 1.6% in 2007/8 and 1.8% in 2017/8, and heart failure at 0.7-0.8% (6). Overall, 7 million people in the UK are estimated to be living with CVD with an estimated annual cost of £19 billion to the UK economy (7).<br><br>Lower respiratory tract infections are also among the global leading causes of death (1). Ecological studies have been used throughout the 20th, and into the 21st, century to demonstrate an excess in CVD incidence and mortality during influenza seasons (8–13). Understanding and addressing interactions between diseases, such as ARIs and CVD, is becoming increasingly important to deal with growing multimorbidity, which can be best tackled through stratified and targeted interventions.<br><br>Pooled estimates from two systematic reviews have showed that the odds of myocardial infarction (MI) are two times higher in those with ILI (14,15). Compared to ILI, the association between pneumonia and acute cardiovascular complications has been less investigated. However, a systematic review conducted in 2010 found that after community acquired pneumonia, inpatients had a pooled incidence of 14.1% (95% CI 9.3–20.6) for acute heart failure and 5.3% (95% CI 3.2–8.6) for ACS (16). Recent studies have also shown an increased rates of MI (17,18) as well as stroke (18) following confirmed influenza or *Streptococcus pneumoniae* infection. Overall less is known about the specific risk of heart failure following ARI (15), although findings from an ecological analysis using the USA Atherosclerosis Risk in Communities Study cohort showed a temporally association between influenza activity and heart failure hospitalisations (19).<br><br>Population studies in the UK, including some which utilised CPRD data, and Canada using a self-controlled case series (SCCS) design have demonstrated that the association between ARI and cardiovascular complications is transient; highest in the first few days after infection (17,18,20,21) but could last for up to one month depending on the infective agent and cardiovascular event (18).<br><br>These existing studies were largely conducted in older populations or those with pre-existing CVD. There is a lack of evidence to determine where cardiovascular complications following ARI exist in those aged less than 65 years and at increased cardiovascular risk but without pre-existing diagnosed CVD. One SCCS analysis of laboratory-confirmed respiratory infections conducted analysis stratified by age group, identifying higher rates of first MI (incidence ratio (IR) 16.1, 95% CI 5.12-50.9) and stroke (IR 23.4, 95% CI 5.71-96.3) in the first three days after a respiratory virus infection in those under 65 years of age (18). The authors hypothesise this is due to lower vaccination rates in the younger population. The number of people included in this younger age group was small producing imprecise estimates. Other studies have not found any increased risk of cardiovascular complications following infection in younger age groups (17,21). These studies were all underpowered to specifically estimate relative incidence and risk in younger age groups.<br><br>In the UK, influenza and pneumococcal vaccination is recommended for all adults aged over 65 years, and people younger than 65 years if they are in a clinical risk group more likely to experience medical complications following an infection, such as those with chronic heart disease (22,23). Influenza and pneumococcal vaccinations are not currently recommended for primary prevention of acute cardiovascular events, such as MI or stroke, in people with raised cardiovascular risk but without established CVD. People with hypertension are considered to be at raised risk of CVD, while the QRISK2 prediction algorithm estimates an individual's 10-year risk of developing CVD based on a wide range of risk factors (24) and is being increasingly used to guide CVD risk assessment and prevention strategies (25).<br><br>This study aims to test the hypothesis that people with raised cardiovascular risk (hypertensive or with calculated QRISK2 ≥10% in 10 years) are at increased risk of cardiovascular events after ARIs compared to those without raised risk. We will produce estimates for this absolute risk to inform future studies which will investigate the impact of influenza and pneumococcal vaccination in these cardiovascular risk groups.
**G. Study Type**<br>Descriptive and hypothesis testing
**H. Study Design**<br>Cohort study
**I. Feasibility counts**

In September 2013, the midpoint of our study period, in CPRD GOLD with linked HES data there were 687,479 patients aged 40-64 years with at least 12 months of research standard follow-up who did not have existing CVD or a chronic condition (respiratory disease, diabetes, liver disease, kidney disease and asplenia or splenic dysfunction) already eligible for influenza or pneumococcal vaccination. Based on previous work conducted by the HPRU in Immunisation (ISAC number 18_218) we estimate a further 1% of patients will be immunosuppressed (26) (and therefore already eligible for vaccination), and assuming a maximum of 5% of patients will additionally receive vaccination (i.e. occupational group or paid for vaccination). This would result in 646,574 patients for inclusion from CPRD GOLD linked data. Using Aurum as well as GOLD we should have twice as large a patient population. We, therefore, estimate that in 2013 using both data source we will have 1,293,148 patients.

Among this population, based on CPRD GOLD with linked HES data:

- Hypertension: we would have 81,382 patients with hypertension at baseline, with a ratio of 1:7 patients with hypertension to those without hypertension.
- QRISK2: we would have 161,811 patients with a cardiovascular risk score recorded at baseline. QRISK2 is only recorded for approximately 10% of eligible patients (27). We also expect a ratio of 2:1 patients for scores <10%:≥10% (28).
- ARIs: we will have 7.7% (n=49,838) of patients with a diagnosis of ARI between September 2013 and August 2014.
- MACE: we will have 0.6% (n=4,035) of patients with a MACE event between September 2013 and August 2014.

J. Sample size considerations

Using the results of our feasibility counts to carry our sample size calculations, we estimate the minimum effect estimates we will be able to detect are:
Exposure group A (hypertension): we can detect hazard ratios of 0.97 for ARIs and 0.91 for MACE (with 80% power and alpha 5%).
Exposure group B (QRISK2): we can detect hazard ratios of 0.97 for ARI and 0.88 for MACE (with 80% power and alpha 5%).

K. Planned use of linked data (if applicable):

ARIs and MACE may be recorded in either primary or secondary care records, with major acute events resulting in hospitalisation. Therefore, to increase ascertainment of these diagnoses we will use CPRD linked to HES in-patient data for our entire study population. To include the outcome of cardiovascular death, ONS mortality data is required to identify these events.

Townsend scores will be used in the calculation of QRISK2 scores. We will additionally use this to consider socioeconomic status as a potential confounder.

The above-mentioned linked data will also be used to identify potential confounders as well, further detail is described in Section N on covariates.

L. Definition of the Study population

The study population is adults aged 40 to 64 in CPRD GOLD or Aurum datasets with linked HES data between 01 September 2008 and 31 August 2018. This time period covers the duration of QRISK2 use. We will start follow up from September as this corresponds to when patients would be assessed for seasonal influenza vaccine eligibility. Our analysis will be divided into influenza/non-influenza season (see Section N for further detail). Only patients with at least 12 months of research standard follow up will be included.

Baseline and follow up are defined for each of the study aims in Section O.

<u>Exclusions</u>

Influenza and pneumococcal vaccines are already offered to a range of risk groups. We aim to identify ARI risk and MACE after ARI risk in patients who are not already eligible for influenza or pneumococcal vaccination. We will therefore exclude at baselines patients who have:

- any previous record for diagnosis of CVD included in either influenza or pneumococcal vaccination policy (chronic heart disease or stroke/TIA).

- any previous record for diagnosis of a chronic condition included in influenza vaccination policy. These include a previous record for diagnosis of; chronic respiratory disease, chronic kidney disease, chronic liver disease, diabetes, asplenia / splenic dysfunction, chronic neurological conditions, or morbid obesity (in years where these were included in influenza vaccination clinical risk groups).
- a record of receiving immunosuppressive treatment (chemotherapy, radiotherapy or immunosuppressive drug prescribed in primary care i.e. steroids) in the year prior to baseline.
- a record of ever receiving pneumococcal vaccine. Previous vaccination could reflect membership of another risk group (beyond those previously outlined) such as an occupational group or uptake of private vaccination, therefore these individuals would be unlikely to benefit further from an extension of the current vaccination recommendations.
- a record of receiving influenza vaccination in the year prior to baseline.

We will define our exclusion groups using codelists created by the HPRU in Immunisation (ISAC protocol 18_218RA).

M. Selection of comparison group(s) or controls

Exposure group A: patients with hypertension will be compared to patients without hypertension.
Exposure group B: patients with a recorded/calculate QRISK2 ≥10% in ten years will be compared to patients with QRISK2 <10%.

N. Exposures, Outcomes and Covariates

Exposures

All analysis will be conducted by cardiovascular risk group defined as:

- Exposure group A (hypertension): patients with a Read or SNOMED code in the GOLD clinical or referral files / Aurum observation (problem or referral) files for a hypertension diagnosis (as listed in Appendix 1 for CPRD GOLD, to be mapped to SNOMED Aurum codes) but without any CVD diagnosis in the same GOLD/Aurum files or ICD-10 code in HES (as defined by HPRU codelists). We will only base our definition of hypertension on recorded diagnosis and not include blood pressure readings or treatments to classify patients into a risk group. This represents the practical method by which patients would be identified in primary care systems to offer vaccination. As a comparison group we will include patients with no hypertension or existing CVD defined as absence of any relevant Read or SNOMED code.
- Exposure group B (raised vascular risk): QRISK2 scores will be calculated based on the previous approach taken by Bhaskaran, Gadd et al (ISAC 17_008). QRISK2 scores will be calculated using bulk processing software provided by ClinRisk, using the most recent measures available. Variables in QRISK2 include age, sex, ethnicity, blood pressure, deprivation score from linked data, diabetes, family history of coronary heart disease, atrial fibrillation, chronic kidney disease stage 4 or 5, cholesterol/HDL ratio, rheumatoid arthritis, use of blood pressure lowering drugs, BMI (using weight and height records), smoking status (using smoking-related Read codes and structured data on smoking). Of note, those with diabetes and chronic kidney disease will be excluded from our study (see Section L Exclusions). Time variant variables such as BMI and smoking status at the most recent date to index date will be utilised and if missing the median value will be assigned. We will also use recorded QRISK2 scores based on Read / SNOMED codes which relate to QRISK in GOLD using the clinical file linked to corresponding results in the additional file and in Aurum using appropriate observation file data. Based on the recorded/calculated score we will group patients into those with a score of ≥10% in ten years who are the group of interest and compared these patients to those with a score of <10% in ten years.

For aim 4, the baseline date will be the date of ARI (see section O for further detail). Patients with a record of a Read or SNOMED code or ICD-10 code in HES for a diagnosis of an ARI as listed in Appendix 2 will be included in follow up.

Outcomes

Aim 1 & 2: the primary outcome will be all-cause acute systemic respiratory infections, with separate secondary outcomes of ILI and pneumonia. Infections will be defined by Read and SNOMED codes or ICD-

10 code in HES as listed in Appendix 2. The codes which will be used to define ILI and pneumonia are also indicated in Appendix 2.

Aim 3 & 4: cardiovascular complications can manifest in many forms. We will use the composite outcome of MACE to account for the wide range of outcomes possible and to achieve statistical power. There is no standard definition of MACE, although most definition include cardiovascular death, MI and stroke. A wider definition often used includes left ventricular heart failure and unstable angina. We will take a broad definition and define MACE as; ACS which will include MI and unstable angina, left ventricular heart failure, stroke, TIA, acute ischaemic limb and cardiovascular death. Outcomes will be defined by Read and SNOMED codes or ICD-10 codes in HES (and ONS for deaths), respectively which are listed in Appendix 3. In sensitivity analysis we will use a narrower definition of MACE with the most severe outcomes; MI, left ventricular heart failure, stroke and cardiovascular death. Our secondary outcomes are each of the cardiovascular conditions separately.

Covariates

We will control for a range of potential confounding variables and investigate potential effect modifiers. We expect, based on existing literature and a priori hypotheses, to include the variables listed below.

- Age: categorised into 5-year bands of 40-44, 45-49, 50-54, 55-59, 60-64 from CPRD Gold/Aurum patient file
- Sex: defined as male or female from CPRD Gold/Aurum patient file
- Ethnicity: from CPRD GOLD/Aurum codes, supplemented with HES data where missing from CPRD and available in HES
- Socioeconomic status: from linked Townsend scores grouped into quintiles
- Consultation frequency: based on in-person and telephone consultations in the year prior to baseline
- Lifestyle factors: alcohol consumption and smoking status from CPRD GOLD additional file as well as codes in the clinical or referral files CPRD / Aurum observation files
- Body Mass Index (BMI): recorded (or if not recorded directly then to be calculated from height and weight recorded) in CPRD GOLD additional file / CPRD Aurum observation files.
- Comorbidities/existing health conditions: those not covered by exclusions such as dementia using Read and SNOMED codes recorded in the CPRD GOLD clinical file / CPRD Aurum observation files.
- Primary prevention of CVD: statins and antihypertensives from CPRD GOLD therapy file / CPRD Aurum observation files.
- Season: when outcome occurs as well as when exposure occurs for aim 3, defined as flu season (1 September – 31 March) or non-flu season (1 April – 31 August).

Effect modifier

- Primary prevention of ARI: influenza and pneumococcal vaccination given in the follow-up year from CPRD GOLD immunisation file / CPRD Aurum observation files.
- Antiviral treatment: if a suitable number of patients have been prescribed antivirals, in stratified analysis we will explore the effect of antiviral use given in the follow-up year both where it is suggested to be for prophylactic use (no ARI diagnosis) and treatment (ARI diagnosis) using data from CPRD GOLD therapy file / CPRD Aurum observation files.
- Antiplatelets: again, if a suitable number of patients have been prescribed an antiplatelet, we will explore their impact in stratified analysis using data from CPRD GOLD therapy file / CPRD Aurum observation files.

O. Data/ Statistical Analysis

To achieve the necessary statistical power we will use both CPRD GOLD and Aurum (query number CPRD00040222). This will require deduplication of data where practices have migrated from Vision to EMIS software system, we will use the file of migrating practices provided by CPRD for this deduplication. Where possible we will arrange GOLD and Aurum datasets into a single combined dataset to analyse individual level data. Where this cannot be achieved, we will analyse data from GOLD and Aurum separately. When analysed separately, to calculate single summary estimates, we will either combine the number of events and person-time from each database (aim 1) or use fixed effects meta-analysis (aim 2-4).

Before calculating single summary estimates between-database heterogeneity (based on the I^2 statistic) will be assessed.

For thorough ascertainment of ARIs and MACE all analyses will be conducted using linked HES data. We will clean the data in order to only count events once. Multiple records in CPRD or HES primary diagnostic position for ARI within 28 days of each other will be counted as the same episode. The index date will be the date of the first ARI consultation and the end of the episode will be 28 days after the last ARI consultation. Confounders where data are missing in CPRD will be identified, where possible, using HES.

Aim 1 (age-specific rates of ARI) and aim 2 (association between cardiovascular risk and ARI rates)
An open cohort of patients without any existing CVD, a chronic clinical condition eligible for vaccination or previous pneumococcal vaccination, and without influenza vaccination or immunosuppression diagnosis/treatment in the year prior to baseline (see exclusions in Section L) will be created. Follow-up will begin at the latest of: 01 September 2008, 40th birthday or 12 months after research standard follow up. Eligibility will be updated during follow-up when new measures become available, with follow up ending at the earliest date of: pneumococcal or influenza vaccination (excluded from further follow up), development of CVD (excluded from further follow up), development of a clinical condition or immunosuppressive state eligible for vaccination (excluded from further follow up), death, transfer out, the practice's last data collection, 65th birthday, or 31 August 2018.

Cardiovascular risk level (hypertension status and then again repeated for QRISK2) will be classified at cohort entry. Hypertension status will be updated when a new diagnosis is made. QRISK2 scores will be updated each time a new measure becomes available or every year (to reflect change in age), whichever is sooner.

For patients meeting inclusion criteria we will describe the baseline characteristics stratified by cardiovascular risk; hypertension compared to no hypertension (exposure group A) and QRISK2 ≥10% compared to QRISK2 <10% (exposure group B). In this we will include the extent and pattern of missing data. Additionally, we will conduct a descriptive analysis of patients who progress from low cardiovascular risk at baseline to high risk in follow up.

Using Poisson regression models, we will estimate the crude annual incidence rates and rate ratios for any ARI (primary outcome) and for ILI and pneumonia (secondary outcomes) which occurred during follow up by each baseline cardiovascular risk groups. Lexis expansion by age group and cardiovascular risk level will be used to estimate stratified incidence rates and rate ratios. Multiple ARI episodes in the same patient will be accounted for by fitting random effects models. Models will be separately produced for exposure group A (hypertension) and exposure group B (QRISK2). We will generate multivariable models which adjust first for age and sex, and then for additional confounders (see section N).

Aim 3 (association between cardiovascular risk and MACE)
Our open cohort of patients meeting inclusion criteria described for aim 1 & 2 will again be followed up from the same baseline. Follow up will end at the earliest date of: MACE (outcome of interest), pneumococcal or influenza vaccination (excluded from further follow up), development of a clinical condition or immunosuppressive state eligible for vaccination (excluded from further follow up), death, transfer out, the practice's last data collection or 31 August 2018.

We will again use Poisson regression with Lexis expansion by age group and cardiovascular risk level, this time to estimate annual stratified incidence rates and rate ratios for the primary and secondary MACE outcomes. We will stratify results by season to identify peaks in incidence and MACE risk. Again, separate models will be produced for exposure group A (hypertension) and exposure group B (QRISK2), with multivariable models first adjusting first for age and sex, and then for additional confounders. If a suitable number of records exist we will also build models which stratify by antiviral and antiplatelet treatments, otherwise sensitivity analysis will be conducted (see below).

Pre-specified sensitivity analyses for aims 1-3

1. Repeat main analysis for years 2015-2017 restricting to patients with recorded QRISK2 score (as opposed to our calculated QRISK2 scores), this will allow consideration of the clinical practice that would have occurred for identifying patients eligible for vaccination had a policy based on QRISK2 score been in place and to compare results obtained with recorded score to those from our calculated scores. We have limited the time period as our algorithm is based on the 2015 version of QRISK (minimal changes to the QRISK2 algorithm occurred between 2015 and 2017).
2. Repeat main analysis only excluding patients who are included in both the current influenza and pneumococcal vaccination eligibility recommendations.
3. Repeat main analysis but replacing the binary season variables for one with four categories; autumn (September-November), winter (December-February), spring (March-May) and summer (June-August).

Pre-specified sensitivity analyses for aim 3

1. Repeat main analysis restricting outcome definition of MACE to only include subset of cardiovascular events (as described in Section N, Outcomes).
2. Repeat main analysis excluding any patients who received antiviral treatment during follow-up. This will only be done if too small a number of patients are vaccinated to allow for meaningful stratified analysis.
3. Repeat sensitivity analysis 2 for antiplatelet treatment.

Aim 4 (association between cardiovascular risk and MACE after ARI)

Start of follow-up will be defined as date of ARI diagnosis. End of follow-up will be the earliest of: MACE diagnosis, death, transfer out, the practice's last data collection, one year after ARI diagnosis date or 31st August 2018. Patients with existing CVD, a chronic clinical condition eligible for vaccination or previous pneumococcal vaccination at the date of ARI will be excluded. Patients with seasonal influenza vaccination or immunosuppression diagnosed within one year of ARI diagnosis date will also be exclude. See exclusions in Section L for further detail.

Cardiovascular risk level (hypertension status and then again repeated for QRISK2) will be classified at cohort entry.

We will use Cox proportional hazards regression to generate hazard ratios for primary and secondary MACE outcomes comparing cardiovascular risk groups (separately for exposure group A and B). Calendar time will be used as the underlying time scale. To account for the same patient having multiple ARIs more than one year apart we will conduct analysis using a random effects model. Models will first be adjusted for age and sex, followed by further confounders. The number of ARI episodes which occurred within the year follow up will included in the models. If a suitable number of records exist we will stratify analysis by vaccination status, antiviral treatment and antiplatelet use, otherwise sensitivity analysis will be conducted (see below).

Pre-specified sensitivity analyses for aim 4

1. Repeat main analysis for years 2015-2017 restricting to patients with recorded QRISK2 score.
2. Repeat main analysis only excluding patients who are included in both the current influenza and pneumococcal vaccination eligibility recommendations.
3. Repeat main analysis but replacing the binary season variables for one with four categories; autumn (September-November), winter (December-February), spring (March-May) and summer (June-August).
4. Repeat main analysis restricting outcome definition of MACE to only include subset of cardiovascular events (as described in Section N, Outcomes).
5. Repeat main analysis excluding any patients who received influenza and/or pneumococcal vaccination during follow-up. This will only be done if too small a number of patients are vaccinated to allow for meaningful stratified analysis.
6. Repeat sensitivity analysis 5 for antiviral treatment.
7. Repeat sensitivity analysis 5 for antiplatelet use.

P. Plan for addressing confounding

We will use multivariable regression to adjust for the potential covariates we have hypothesised (listed in section N).

Q. Plans for addressing missing data

We will describe all missing data and consider findings as a limitation in any outputs resulting from our study.

Missing data on acute systemic respiratory infection: People who experience mild and short-lived respiratory illness are unlikely to seek healthcare, this would lead to us underestimating not only the incidence of ARIs but also their association with cardiovascular outcomes. We are interested in severe infections, for which it is more biologically plausible to have systemic complications, which are more likely to result in healthcare attendance. However, we will first conduct our analysis (aim 3) without the inclusion of ARIs to analyse the effect of cardiovascular risk on MACE stratifying by season to determine whether the pattern of MACE follows the seasonality of some ARIs i.e. ILI. Patients who regularly attend their GP may be more likely to present with an ARI, we will therefore include consultation frequency in our models where this is identified as a confounder.

Missing data on cardiovascular outcomes: We will use HES data to supplement primary care recording of all cardiovascular events with our analysis limited to linked data.

Missing data on the classification of cardiovascular risk: While blood pressure and therefore hypertension are generally well recorded in primary care data this requires patients to have presented to the GP. For patients who do not often attend the GP the diagnosis will not be made and therefore those with hypertension recorded may be healthier with better managed hypertension. The algorithm to calculate QRISK2 score is based on many variables. Missing data for the chronic conditions included in the algorithm indicates that the patient does not have the risk factor in question although, similar to hypertension, there may be undiagnosed or under-recording. Missing data for variables such as ethnicity, BMI, alcohol intake and smoking status are due to non-recording. HES will be used to increase the ascertainment of ethnicity. Our QRISK2 algorithm will replace missing values for alcohol intake, smoking status and BMI with the median value, in line with the QRISK2 calculator. We will compare our calculated QRISK2 scores to those recorded by GPs to validate our classifications and conduct a sensitivity analysis which only includes patients with a recorded QRISK2 score. Our algorithm to calculate QRISK2 scores is based on the 2015 version of QRISK2, we will therefore only include patients with QRISK2 score which were recorded in 2015-2017 (during which time changes to the QRISK2 algorithm were minimal).

Missing data on covariates (such as smoking status, alcohol intake, BMI and ethnicity): as data are unlikely to be missing at random i.e. the assumption required for multiple imputation to be valid, we will conduct complete case analysis for variables not set by the presence or absence of a code.

R. Patient or user group involvement (if applicable)

Patients and user groups have not been involved in the development of this research.

S. Plans for disseminating and communicating study results, including the presence or absence of any restrictions on the extent and timing of publication

The study findings will be submitted for publication in a peer-reviewed scientific journal. Additionally, results will be presented at conferences and other meetings as appropriate. We will work with the LSHTM press office to publicise our study and its findings. The electronic health records research group at LSHTM also have a webpage with research news and social media accounts which will be used. We will work with the British Heart Foundation, who are the funders of this research, to publicise our findings to a wider audience including the general public and patient groups.

Conflict of interest statement: The work is supported by a British Heart Foundation PhD Studentship (FS/18/71/33938). There are no conflicts of interest to declare

T. Limitations of the study design, data sources, and analytic methods

As discussed in Section Q on missing data, there is a risk of misclassifying whether patients have had an ARI when using routinely healthcare records data as a large number of people will not attend healthcare for this, particularly those with mild or short-lived illness. Those who do attend are likely to be patients who

have higher rates of consultation overall and those with underlying illness. We have outlined our plan to address this in Section Q.

There is also a risk of misclassification of MACE, however previous validation of CPRD and HES for MI has identified these sources to be of high quality, with positive predictive values of >90% in each (29).

Missing data, particularly for some covariates to be included in models, presents a limitation to our analysis and therefore interpretation of our results. As described in Section Q, we will describe missing data and conduct complete case analysis.

As our analysis aims to estimate the effect of ARI and subsequent MACE which could be avoided by vaccination, we are excluding many groups who are already eligible for vaccination under existing policy. This relies on the ability to identify eligible populations, we will exclude major risk groups with chronic lung disease, chronic kidney disease, chronic liver disease, diabetes and neurological conditions. There may be smaller risk groups which we have missed. We will stratify for vaccination which occurs during follow-up. However, if vaccination was conducted outside of primary care, for example if given through occupational health or paid for at a pharmacy, then we may not be able to reliably identify these unless GPs record the vaccine as having been provided by other healthcare provider.

U. References

1. World Health Organization. The top 10 causes of death [Internet]. Available from: https://www.who.int/en/news-room/fact-sheets/detail/the-top-10-causes-of-death
2. GBD 2016 Stroke Collaborators CO, Nguyen M, Roth GA, Nichols E, Alam T, Abate D, et al. Global, regional, and national burden of stroke, 1990-2016: a systematic analysis for the Global Burden of Disease Study 2016. Lancet Neurol. 2019 Mar 11;0(0).
3. Evers SMAA, Struijs JN, Ament AJHA, van Genugten MLL, Jager J (Hans) C, van den Bos GAM. International Comparison of Stroke Cost Studies. Stroke. 2004 May 1;35(5):1209–15.
4. Saka O, McGuire A, Wolfe C. Cost of stroke in the United Kingdom. Age Ageing. 2008 Nov 13;38(1):27–32.
5. Chevreul K, Durand-Zaleski I, Gouépo A, Fery-Lemonnier E, Hommel M, Woimant F. Cost of stroke in France. Eur J Neurol. 2013 Jul 1;20(7):1094–100.
6. British Heart Foundation. Cardiovascular Disease Statistics 2019 [Internet]. 2019. Available from: https://www.bhf.org.uk/what-we-do/our-research/heart-statistics/heart-statistics-publications/cardiovascular-disease-statistics-2018
7. British Heart Foundation. Heart statistics publications BHF Statistics Factsheet UK [Internet]. 2018. Available from: https://www.bhf.org.uk/what-we-do/our-research/heart-statistics/heart-statistics-publications
8. Collins SD. Excess Mortality from Causes Other than Influenza and Pneumonia during Influenza Epidemics. Public Heal Reports. 1932;47(46):2159.
9. Tillet HE, Smith JWG, Gooch CD. Excess Deaths Attributable to Influenza in England and Wales: Age at Death and Certified Cause. Int J Epidemiol. 1983 Sep 1;12(3):344–52.
10. Reichert TA, Simonsen L, Sharma A, Pardo SA, Fedson DS, Miller MA. Influenza and the Winter Increase in Mortality in the United States, 1959-1999. Am J Epidemiol. 2004 Sep 1;160(5):492–502.
11. Warren-Gash C, Bhaskaran K, Hayward A, Leung GM, Lo S-V, Wong C-M, et al. Circulating Influenza Virus, Climatic Factors, and Acute Myocardial Infarction: A Time Series Study in England and Wales and Hong Kong. J Infect Dis. 2011 Jun 15;203(12):1710–8.
12. Nguyen JL, Yang W, Ito K, Matte TD, Shaman J, Kinney PL. Seasonal Influenza Infections and Cardiovascular Disease Mortality. JAMA Cardiol. 2016 Jun 1;1(3):274.
13. Blackburn R, Zhao H, Pebody R, Hayward A, Warren-Gash C. Laboratory-Confirmed Respiratory Infections as Predictors of Hospital Admission for Myocardial Infarction and Stroke: Time-Series Analysis of English Data for 2004–2015. Clin Infect Dis. 2018 Jun 18;67(1):8–17.
14. Barnes M, Heywood AE, Mahimbo A, Rahman B, Newall AT, Macintyre CR. Acute myocardial infarction and influenza: a meta-analysis of case–control studies. Heart. 2015 Nov 1;101(21):1738–47.

15. Kwok CS, Aslam S, Kontopantelis E, Myint PK, Zaman MJS, Buchan I, et al. Influenza, influenza-like symptoms and their association with cardiovascular risks: a systematic review and meta-analysis of observational studies. Int J Clin Pract. 2015 Sep;69(9):928–37.
16. Corrales-Medina VF, Suh KN, Rose G, Chirinos JA, Doucette S, Cameron DW, et al. Cardiac Complications in Patients with Community-Acquired Pneumonia: A Systematic Review and Meta-Analysis of Observational Studies.
17. Kwong JC, Schwartz KL, Campitelli MA, Chung H, Crowcroft NS, Karnauchow T, et al. Acute Myocardial Infarction after Laboratory-Confirmed Influenza Infection. N Engl J Med. 2018 Jan 25;378(4):345–53.
18. Warren-Gash C, Blackburn R, Whitaker H, McMenamin J, Hayward AC. Laboratory-confirmed respiratory infections as triggers for acute myocardial infarction and stroke: a self-controlled case series analysis of national linked datasets from Scotland. Eur Respir J. 2018 Mar;51(3):1701794.
19. Kytömaa S, Hegde S, Claggett B, Udell JA, Rosamond W, Temte J, et al. Association of Influenza-like Illness Activity With Hospitalizations for Heart Failure. JAMA Cardiol. 2019 Apr 1;4(4):363.
20. Smeeth L, Thomas SL, Hall AJ, Hubbard R, Farrington P, Vallance P. Risk of myocardial infarction and stroke after acute infection or vaccination. N Engl J Med. 2004 Dec 16;351(25):2611–8.
21. Warren-Gash C, Hayward AC, Hemingway H, Denaxas S, Thomas SL, Timmis AD, et al. Influenza Infection and Risk of Acute Myocardial Infarction in England and Wales: A CALIBER Self-Controlled Case Series Study. J Infect Dis. 2012 Dec 1;206(11):1652–9.
22. Public Health England. Influenza: the green book, chapter 19 [Internet]. 2019. Available from: https://www.gov.uk/government/publications/influenza-the-green-book-chapter-19
23. Public Health England. Pneumococcal: the green book, chapter 25 [Internet]. 2018. Available from: https://www.gov.uk/government/publications/pneumococcal-the-green-book-chapter-25
24. Hippisley-Cox J, Coupland C, Brindle P. Development and validation of QRISK3 risk prediction algorithms to estimate future risk of cardiovascular disease: prospective cohort study. BMJ. 2017 May 23;357:j2099.
25. National Institute for Health and Care Excellence. CVD risk assessment and management [Internet]. 2019.
26. Grint D, Thomas SL, Evans D, Walker JL, Andrews NJ, Tessier E, et al. What can research primary care datasets contribute to routine monitoring of vaccine coverage and uptake? In Public Health Research & Science Conference, Manchester UK.; 2019.
27. Finnikin S, Ryan R, Marshall T. Statin initiations and QRISK2 scoring in UK general practice: a THIN database study. Br J Gen Pract. 2017 Dec;67(665):e881–7.
28. Robson J, Dostal I, Sheikh A, Eldridge S, Madurasinghe V, Griffiths C, et al. The NHS Health Check in England: an evaluation of the first 4 years. BMJ Open. 2016 Jan 13;6(1):e008840.
29. Herrett E, Shah AD, Boggon R, Denaxas S, Smeeth L, van Staa T, et al. Completeness and diagnostic validity of recording acute myocardial infarction events in primary care, hospital care, disease registry, and national mortality records: cohort study. BMJ. 2013 May 21;346(may20 3):f2350–f2350.
